# Several Indicators of Critical Transitions for Complex Diseases Based on Stochastic Analysis

**DOI:** 10.1155/2017/7560758

**Published:** 2017-08-01

**Authors:** Gang Wang, Yuanyuan Li, Xiufen Zou

**Affiliations:** ^1^School of Mathematics and Statistics, Wuhan University, Wuhan 430072, China; ^2^Computational Science Hubei Key Laboratory, Wuhan University, Wuhan 430072, China

## Abstract

Many complex diseases (chronic disease onset, development and differentiation, self-assembly, etc.) are reminiscent of phase transitions in a dynamical system: quantitative changes accumulate largely unnoticed until a critical threshold is reached, which causes abrupt qualitative changes of the system. Understanding such nonlinear behaviors is critical to dissect the multiple genetic/environmental factors that together shape the genetic and physiological landscape underlying basic biological functions and to identify the key driving molecules. Based on stochastic differential equation (SDE) model, we theoretically derive three statistical indicators, that is, coefficient of variation (CV), transformed Pearson's correlation coefficient (TPC), and transformed probability distribution (TPD), to identify critical transitions and detect the early-warning signals of the phase transition in complex diseases. To verify the effectiveness of these early-warning indexes, we use high-throughput data for three complex diseases, including influenza caused by either H3N2 or H1N1 and acute lung injury, to extract the dynamical network biomarkers (DNBs) responsible for catastrophic transition into the disease state from predisease state. The numerical results indicate that the derived indicators provide a data-based quantitative analysis for early-warning signals for critical transitions in complex diseases or other dynamical systems.

## 1. Introduction 

A sudden change of a system is a recurrent phenomenon in many complex diseases, which often occurs at a critical threshold, or the so-called “tipping point,” and can be interpreted as the fact that the system shifts abruptly from one asymptotically stable equilibrium to another one [1]. During the progression of many complex diseases, for example, in chronical diseases such as cancer, the deterioration is abrupt [2, 3]. In other words, there exists a sudden catastrophic shift during the progress of gradual health deterioration that results in a drastic transition to a disease state. Intuitively, between the healthy state and the disease state, there is a critical state which is the so-called “critical transition” [4, 5]. Therefore, in recent years, there has been a growing interest in developing quantitative and qualitative approaches for detecting early-warning signals to avoid such undesirable transitions. The model-based and network-based approaches, respectively, have been used to extract warning signals from observed time series. Therefore, a variety of empirical studies based on analysis of time-series data have suggested that some statistical signatures, such as variance, Pearson's correlation coefficient (PCC), autocorrelation, and coefficient of variation (CV), may be used to predict the critical transition [6–14]. To extend and generalize the proposed indicators and find new statistical characteristics, in this study, we use a generic stochastic differential equation (SDE) model to represent the complex system based on time-series data with noise and try to understand the critical transition from a mathematical viewpoint. Combining the qualitative theory of fast-slow dynamical systems, probability theory, and statistical analysis, we theoretically prove that three statistical indicators, that is, coefficient of variation (CV), transformed Pearson's correlation coefficient (TPC), and transformed probability distribution (TPD), can distinguish the early-warning signals of the critical transition in complex systems. Finally, we use real high-throughput data for three complex diseases, including influenza caused by either H3N2 virus [15] or H1N1 virus [16] and acute lung injury induced by carbonyl chloride inhalation exposure [17], to demonstrate the applicability of these early-warning indexes for extracting the dynamical network biomarkers (DNBs) responsible for catastrophic transition into the disease state from predisease state.

## 2. Methods

### 2.1. Preliminaries for Dynamical Systems and Stochastic Differential Equations

The dynamics for the progression of a complex system can be expressed by the following nonlinear continuous-time equations (ODEs):

(1) $$\begin{array}{c} \frac{dZ\left(t\right)}{dt}=f\left(\;Z\left(\;t\;\right);P\;\right), \end{array}$$

where *Z*(*t*) = (*z*_1_(*t*), *z*_2_(*t*),…,z_*n*_(*t*))^*T*^ ∈ *ℝ* is an *n*-dimensional state vector at time instant *t* with *t* ∈ *ℝ*_+_ and *P* = (*p*_1_, *p*_2_,…,*p*_*s*_)^*T*^ ∈ *ℝ*^*s*^ is a *s*-dimensional parameter vector or driving factors. *f* : *ℝ*^*n*^ × *ℝ*^*s*^ → *ℝ*^*n*^ is an *n*-dimensional nonlinear function vector. Suppose that the bifurcation of the nonlinear dynamical system occurs at the bifurcation point (*Z*^*∗*^, *P*^*∗*^), where *Z*^*∗*^ = (*z*_1_^*∗*^, *z*_2_^*∗*^,…,*z*_*n*_^*∗*^)^*T*^ and *P*^*∗*^ = (*p*_1_^*∗*^, *p*_2_^*∗*^,…,*p*_*s*_^*∗*^)^*T*^. For system (1) near *Z*^*∗*^, before *P* reaches *P*^*∗*^, the system is supposed to stay at an asymptotically stable equilibrium *Z*^*∗*^ and therefore all the real parts of the eigenvalues are negative [18, 19].

Generally, there exist the noises in the real system [20, 21]. Thus, we add the white Gaussian noises to the original ordinary differential system (1) and obtain the following stochastic differential equations:

(2) $$\begin{array}{c} \frac{dZ\left(t\right)}{dt}=f\left(\;Z\left(\;t\;\right);P\;\right)+H\frac{dB\left(t\right)}{dt}, \end{array}$$

where *B*(*t*) = (*B*_1_(*t*), *B*_2_(*t*),…,*B*_*m*_(*t*))^*T*^ are *m*-dimensional standard Brownian motion.

Consider the linearized equations of (1) with the perturbations of white Gaussian noises near *Z*^*∗*^. Namely, introducing new variables *Y*(*t*) = (*y*_1_(*t*), *y*_2_(*t*),…,*y*_*n*_(*t*))^*T*^ and a transformation matrix *S*, that is, *Y*(*t*) = *S*^−1^(*Z*(*t*) − *Z*^*∗*^) (see the detailed process in Supplementary Information A1 in the Supplementary Material available online at https://doi.org/10.1155/2017/7560758), we have

(3) $$\begin{array}{c} \frac{dY\left(t\right)}{dt}=\Lambda \left(\;P\;\right)Y\left(\;t\;\right)+G\frac{dB\left(t\right)}{dt}, \end{array}$$

where Λ(*P*) is a diagonal matrix which is the Jordan normal form for the Jacobian matrix (∂*f*(*Z*; *P*)/∂*Z*)|_*Z*=*Z*^*∗*^_ and

(4) $$\begin{array}{c} G=\left(\begin{array}{cccc} g_{11} & g_{12} & \cdots & g_{1m}\\ g_{21} & g_{22} & \cdots & g_{2m}\\ \vdots & \vdots & & \vdots \\ g_{n1} & g_{n2} & \cdots & g_{nm} \end{array}\right)\in R^{n\times m}, \end{array}$$

with

(5) $$\begin{array}{c} \overset{m}{\underset{l=1}{\sum }}g_{il}g_{jl}\neq 0,\;i,j=1,2,\ldots ,n. \end{array}$$

Actually, there are typically three cases arising in diagonal matrix Λ(*P*). For the sake of simplicity, here we only illustrate case  1, that is, Λ(*P*) = diag⁡(*λ*_1_(*P*), *λ*_2_(*P*),…, *λ*_*n*_(*P*)) with each *λ*_*i*_ negative. The other two cases are described in Supplementary Information A1: a generic model in abstract phase space and some preliminaries.

Among the eigenvalues of Λ in case 1, the largest one, say *λ*_1_, first approaches 0 when parameter *P* → *P*^*∗*^. This eigenvalue characterizes the rate of the system around the steady state approximately and is called the dominant eigenvalue. We can calculate the solution of the stochastic differential equation (3):

(6) yit=eλit−t0yit0+∑k=1mgik∫t0teλit−t0dBks,i=1,2,…,n.

It is easy to obtain that

(7) Eyit=eλit−t0yit0,var⁡yit=∑k=1mgik21−e2λit−t0−2λi,cov⁡yit,yjt=∑k=1mgikgjk1−eλi+λjt−t0−λi+λj,i,j=1,2,…,n.

Here when the dominant eigenvalue *λ*_1_ → 0^−^ because of the change in the parameter values, var⁡(*y*_1_) → +*∞*, but var⁡(*y*_*i*_) is bounded for *i* ≠ 1 because *λ*_*i*_ < *λ*_1_ < 0, (*i* = 2,…, *n*). For any *i* ≠ *j*, cov⁡(*y*_*i*_, *y*_*j*_) tends to have a positive bounded value.

Returning to the original variables *Z* whose elements can be expressed by *z*_*i*_ = ∑_*j*=1_^*n*^*s*_*ij*_*y*_*j*_ + *z*_*i*_^*∗*^, where *s*_*ij*_ is the element of the linear transformation *S*, we have (Supplementary Information A2)

(8) Ezi=∑j=1nsijEyj+zi∗,cov⁡zi,zj=si1sj1var⁡y1+⋯+sinsjnvar⁡yn+∑k=1  n∑m=1,m≠knsiksjmcov⁡yk,ym,PCCzi,zj=cov⁡zi,zjvar⁡zivar⁡zj.

Notice that variable *y*_1_ is related to the dominant eigenvalue *λ*_1_. From the above equations, it is obvious that as *λ*_1_ → 0^−^, the variance, that is, var⁡(*z*_*i*_) = cov⁡(*z*_*i*_, *z*_*j*_), increases greatly, or var⁡(*z*_*i*_) → +*∞* if *s*_*i*1_ is not vanishing, and |PCC(*z*_*i*_, *z*_*j*_)| approaches 1 drastically if both *s*_*i*1_ and *s*_*j*1_ are nonzero. In this case, variables *z*_*i*_ and *z*_*j*_ are directly affected by the dominant eigenvalue. A group composed of such variables is called the dominant group in the network. On the other hand, as *λ*_1_ → 0^−^, |PCC(*z*_*i*_, *z*_*j*_)| between the dominant group (e.g., including *z*_*i*_) and others (e.g., including *z*_*j*_, which does not belong to the dominant group) reduces to zero if *s*_*i*1_ ≠ 0 but *s*_*j*1_ = 0.

In addition, from dynamical viewpoint, a system that leads to critical transition is due to the change of the dominant eigenvalue. This viewpoint can be understood from the process of the theoretical derivation in Supplementary Information A1. In other words, the fact that the eigenvalue approaches zero when the critical transition occurs is very important. Simultaneously, a very necessary question arises: how can we describe the concept “related to the dominant eigenvalue”? In order to achieve our goal, using the theory of the Taylor expansion and the Jordan normal form of matrix, the key point is that the model can be projected onto the center manifold under the linear transformation. Then by identifying the coefficient with respect to the dominant eigenvalue being zero or not, we can determine which group of variables can lead to critical transition. Therefore, all the variables *z*_*i*_ with *s*_*i*1_ ≠ 0 form a group, which indicates that a transition from a steady state to another steady state will occur. It is easy to see that this group characterizes the statistical features of the underlying system. Especially in complex diseases, the molecules in this group are strongly and dynamically correlated in the predisease state (see Section 2.3), which can be viewed as DNBs for early-warning signals.

### 2.2. Method for Identifying Dynamical Network Biomarkers (DNBs) for Complex Diseases Based on Real Data

The evolutionary process of the complex diseases can be generally categorized into three states: that is, a normal state, a predisease state, and a disease state [20–25]. The normal state is a steady state that represents a relatively healthy stage during which the disease is under control. The predisease state corresponds to the critical state before transition to the disease state. In this stage, it may be reversible to the healthy stage under appropriate and therapeutic interventions [26]. Therefore, it is of great importance to identify DNBs representing an early-warning signals of disease progression, or as a leading biomarker that drives the system into the disease state. In this paper, the identification of DNBs includes four steps. For a given real dataset, the first step is to calculate the indicators CV and TPD of each protein in the symptomatic group and the asymptomatic group, respectively. Then, in second step, the* t*-test method is used to select the significantly different proteins of the indicators CV and TPD between symptomatic and asymptomatic groups as candidates. In third step, we choose the intersection of candidate of indicators CV and TPD. In final step, the proposed TPC is calculated for above candidates and the distinguished DNBs are selected.

### 2.3. Data Collection

Three gene expressing profiling datasets were downloaded from the NCBI GEO database (GSE30550, GSE52428, and GSE2565). Probe sets from these datasets lacking the corresponding gene symbols were ignored in our analysis. The expression values of the probe sets that mapped to the same gene were averaged. The diseases from the first two datasets were two influenza strains, H3N2 and H1N1, whereas the other one dataset was for acute lung injury.

The biological data GSE3055024 contained 17 healthy subjects who received intranasal inoculations of influenza H3N2/Wisconsin [15]. Nine of these 17 subjects developed severe infection symptoms, and the other 8 subjects remained in good health. Gene expression profiles were measured in whole peripheral blood drawn from all subjects approximately every 8 hours postinoculation (hpi) through 108 hpi. In total, 268 gene expression profiles were obtained for all subjects at 16 time points, including baseline (−24 hpi). The gene expression profiles of subject 8 at 21 hpi, subject 13 at baseline and 36 hpi, and subject 17 at 36 hpi were missing.

The biological dataset GSE5242825 contained 24 healthy subjects who received intranasal inoculations of influenza H1N1/Brisbane [16]. 12 of these 24 subjects developed severe infection symptoms and 11 subjects remained in good health. One subject was excluded from all analyses because the symptoms began late and were thought to be related to infection acquired in the facility rather than from the primary infection related to inoculation. Gene expression profiles were measured as described for the biological dataset GSE30550.

The biological dataset GSE256526 contained 6 control samples (control group) and 6 case samples (case group) [17]. CD-1 male mice were divided into two groups that were exposed to air or phosgene. Lung tissues were collected from air- or phosgene-exposed mice at 0.5, 1, 4, 8, 12, 24, 48, and 72 hours (h) after exposure.

## 3. Results

### 3.1. Statistical Characteristics near Critical Phase Transition

In this paper, we focus on the following three statistical indexes.

For the convenience of the following statements, we will introduce some symbols and equations mentioned in the “Methods”; that is,

(9) $$\begin{array}{c} \frac{dY\left(t\right)}{dt}=\Lambda \left(\;P\;\right)Y\left(\;t\;\right)+G\frac{dB\left(t\right)}{dt}, \end{array}$$

where *Y*(*t*) = (*y*_1_(*t*), *y*_2_(*t*),…,*y*_*n*_(*t*))^*n*^ ∈ *ℝ*^*n*^, Λ(*P*) ∈ *ℝ*^*n*×*n*^, *G* ∈ *ℝ*^*n*×*m*^, and *B*(*t*) = (*B*_1_(*t*), *B*_2_(*t*),…,*B*_*m*_(*t*))^*T*^ are *m*-dimensional standard Brownian motion.

In addition,

(10) $$\begin{array}{c} z_{i}\left(\;t\;\right)=\overset{n}{\underset{j=1}{\sum }}s_{ij}y_{j}\left(\;t\;\right)+z_{i}^{*},\;i=1,2,\ldots ,n, \end{array}$$

where

(11) Zt=z1t,z2t,…,zntT∈Rn,Z∗=z1∗,z2∗,…,zn∗T∈Rn,P=p1,p2,…,psT∈Rs,P∗=p1∗,p2∗,…,ps∗T∈Rs,S=sijn×n∈Rn×n.

(*1) The Coefficient of Variation CV*. For a given variable *X*, we have the following equation for CV:

(12) $$\begin{array}{c} CV\left(\;X\;\right)=\frac{\sqrt{\mathrm{var}\left(X\right)}}{E\left(X\right)}, \end{array}$$

where var⁡(*X*) and *E*(*X*) are the variance and mean value for *X*, respectively.

(*2) Transformed Pearson's Coefficient (TPC)*. As we know, −1 ≤ PCC(*X*, *Y*) ≤ 1, where PCC(*X*, *Y*) is Pearson's correlation coefficient (PCC) between the random variables *X* and *Y*. To make it more effective in practice, we can define an indicator with respect to PCC as follows:

(13) $$\begin{array}{c} TPC\left(\;X,Y\;\right)=-\ln\left(\;1-\left|\;PCC\left(\;X,Y\;\right)\;\right|\;\right). \end{array}$$

(*3) Transformed Probability Distribution (TPD)*. Obviously,

(14) $$\begin{array}{c} z_{i}\left(\;t\;\right)\sim N\left(\;\mu_{i}\left(\;t\;\right),{\left(\;\sigma_{i}\left(\;t\;\right)\;\right)}^{2}\;\right),\;i=1,2,\ldots ,n, \end{array}$$

where

(15) μit=Ezit,σit=var⁡zit.

Thus, the probability density of the random variable *z*_*i*_(*t*) is

(16) $$\begin{array}{c} p_{i}\left(\;x\;\right)=\frac{1}{\sqrt{2\pi }\sigma_{i}}e^{-\left(x-\mu_{i}\right)/2\sigma_{i}^{2}}. \end{array}$$

Then, we can define the indicator TPD as

(17) $$\begin{array}{c} {TPD}_{i}\left(\;\left[\;a,b\;\right]\;\right)=-\ln\left(\;\overset{b}{\underset{a}{\int }}p_{i}\left(\;s\;\right)ds\;\right), \end{array}$$

where 0 < *a* < *b*.

The theoretical results for these indicators are presented in parts below.

*Coefficient of Variation (CV)*. According to the definition of CV, we can consider case  1 described in Supplementary Information A1 for which Λ is diagonal.

**Theorem 1 .**

Suppose that Λ in (9) is diagonal; then

(18) $$\begin{array}{c} \underset{t\to +\infty \;}{lim}\underset{P\to P^{*}}{lim}CV\left(\;z_{i}\left(\;t\;\right)\;\right)=\left\{\begin{array}{cc} +\infty & s_{i1}\neq 0,\\ A\;\;finite\;\;number & s_{i1}=0. \end{array}\right. \end{array}$$

**Proof**

A detailed proof is given in Supplementary Information  A2.1.  

We recall that *z*_*i*_(*t*) = ∑_*j*=1_^*n*^*s*_*ij*_*y*_*j*_(*t*) + *z*_*i*_^*∗*^,   *i* = 1,2,…, *n*. *s*_*i*1_ ≠ 0 implies that *z*_*i*_ = ∑_*l*=1_^*n*^*s*_*il*_*y*_*l*_(*t*) + *z*_*i*_^*∗*^ is related to the dominant eigenvalue. *s*_*i*_1_1_ ≠ 0 and *s*_*i*_2_1_ = 0 mean that *z*_*i*_1__ = ∑_*l*=1_^*n*^*s*_*i*_1_*l*_*y*_*l*_(*t*) + *z*_*i*_1__^*∗*^ may lead to the critical transition and *z*_*i*_2__ = ∑_*l*=2_^*n*^*s*_*i*_2_*l*_*y*_*l*_(*t*) + *z*_*i*_2__^*∗*^ may not lead to the critical transition. For the convenience to discuss in the following sections, we denote *z*_*i*_1__(*t*) and *z*_*i*_2__(*t*) as a biomarker and a nonbiomarker, respectively. Then, we can prove the following corollary.

**Corollary 2 .**

As the parameter *P* approaches the bifurcation value *P*^*∗*^, that is, the system is close to the critical transition,

1. there are no drastic changes for the coefficient of variation CV of nonbiomarkers;
2. the coefficient of variation CV for biomarkers is much larger than the coefficient of variation CV for nonbiomarkers;
3. the coefficient of variation CV for biomarkers drastically increases.

**Proof**

A detailed  proof is given in Supplementary Information A2.1.  

*Transformed Pearson's Correlation Coefficient (TPC)*. According to the definition of TPC, we can derive the following theorem.

**Theorem 3 .**

Suppose that Λ in (9) is diagonal; then

(19) limt→+∞ limP→P∗TPCzit,zjt=+∞si1≠0,  sj1=0,0si1=0,  sj1=0,A  finite  numbersi1=0,  sj1=0.

**Proof**

A detailed proof is given in Supplementary Information A2.2.

Based on Theorem 3, we can prove the following corollary.

**Corollary 4 .**

As the parameter *P* approaches the bifurcation value *P*^*∗*^,

1. there are no drastic changes for the indicator TPC between nonbiomarkers;
2. the indicator TPC between biomarkers is much larger than TPC between nonbiomarkers;
3. the indicator TPC between a biomarker and a nonbiomarker is much smaller than TPC between nonbiomarkers;
4. the indicator TPC between biomarkers drastically increases.

**Proof**

A detailed proof is given in Supplementary Information A2.2. 

*Transformed Probability Distribution (TPD)*. According to the definition of TPD, we have Theorem 5.

**Theorem 5 .**

Suppose that Λ in (9) is diagonal; then

(20) limt→+∞ limP→P∗TPDia,b=+∞si1≠0,A  finite  numbersi1=0,

where 0 < *a* < *b*.

**Proof**

A detailed proof is given in Supplementary Information A2.3.

Similarly, we can easily obtain the corresponding corollary.

**Corollary 6 .**

As the parameter *P* approaches the bifurcation value *P*^*∗*^,

1. there are no drastic changes for the indicator TPD of nonbiomarkers;
2. the indicator TPD for biomarkers is much larger than TPD for nonbiomarkers;
3. the indicator TPD for biomarkers drastically increases.

### 3.2. Results for Predicting DNBs in Real Datasets for Complex Diseases

Three real datasets for complex diseases, that is, H3N2, H1N1, and acute lung injury, respectively, are used to illustrate the above theoretical results. According to the steps proposed in the above part “Method for Identifying Dynamical Network Biomarkers (DNBs) for Complex Diseases Based on Real Data,” we calculate three indicators CV, TPD, and TPC for these datasets. The obtained DNBs are listed in Table 1. The second column *number*  *for* CV and third column *number*  *for* TPD represent the numbers of proteins chosen by CV and TPD, respectively. The last column denotes the final selected DNBs by combining CV, TPC, and TPD. In the following, we only present the application on the dataset of the complex disease H3N2 as an example. The detailed descriptions of the results on the other two datasets H1N1 and lung are given in Figures  S1–S9 and Figures  S10–S17 in Supplementary Information B, respectively.

**Figure 1:**
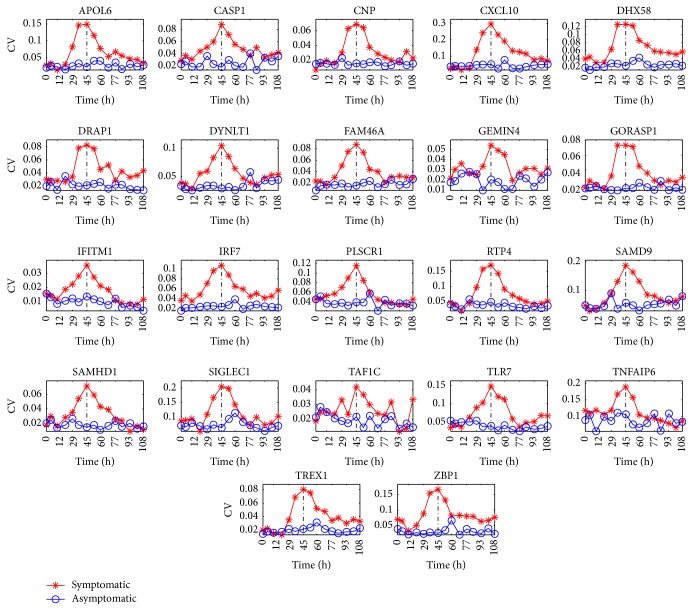
The change of the coefficient of variation CV for 22 proteins: APOL6, CASP1, CNP, CXCL10, DHX58, DRAP1, DYNLT1, FAM46A, GEMIN4, GORASP1, IFITM1, IRF7, PLSCR1, RTP4, SAMD9, SAMHD1, SIGLEC1, TAF1C, TLR7, TNFAIP6, TREX1, and ZBP1. The *x*-axis denotes the time (unit: h). It indicates that as the critical transition occurs, that is, the time evolves towards *t* = 45 h (see the vertical black line), the coefficient of variation CV for 22 proteins above significantly increases in the symptomatic group and has no obvious change in the asymptomatic group. Therefore, these 22 proteins can be viewed as DNBs for early-warning signals.

**Figure 2:**
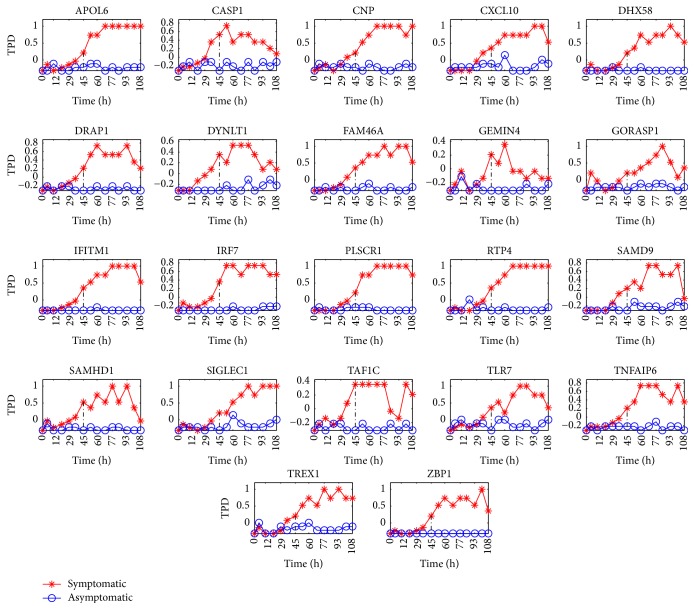
The change of the indicator TPD for 22 proteins: APOL6, CASP1, CNP, CXCL10, DHX58, DRAP1, DYNLT1, FAM46A, GEMIN4, GORASP1, IFITM1, IRF7, PLSCR1, RTP4, SAMD9, SAMHD1, SIGLEC1, TAF1C, TLR7, TNFAIP6, TREX1, and ZBP1. The *x*-axis denotes the time (unit: h). It indicates that as the critical transition occurs, that is, the time evolves towards *t* = 45 h (see the vertical black line), the indicator TPD for 22 proteins above significantly increases. Therefore, these 22 proteins can be viewed as DNBs for early-warning signals.

To further present the results for the H3N2, the change curves of two indicators CV and TPD for the chosen 22 proteins are depicted in Figures 1 and 2, respectively. From these two figures, we can observe that as the critical transition occurs, that is, the time evolves towards *t* = 45 h (see the vertical black line), the CV and TPD for 22 proteins above are significantly increasing in the symptomatic group. But they have no obvious change in the asymptomatic group. Therefore, these 22 proteins can be viewed as DNBs for early-warning signals.

**Figure 3:**
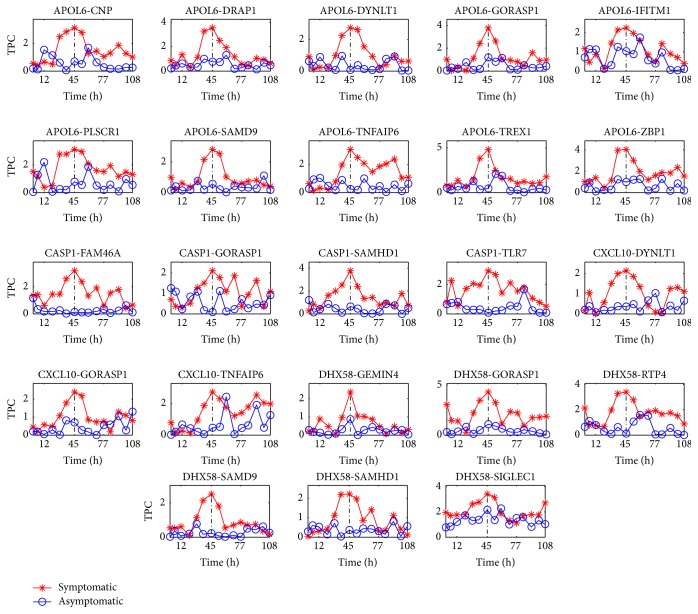
The change of the indicator TPC for 23 protein pairs: APOL6-CNP, APOL6-DRAP1, APOL6-DYNLT1, APOL6-GORASP1, APOL6-IFITM1, APOL6-PLSCR1, APOL6-SAMD9, APOL6-TNFAIP6, APOL6-TREX1, APOL6-ZBP1, CASP1-FAM46A, CASP1-GORASP1, CASP1-SAMHD1, CASP1-TLR7, CXCL10-DYNLT1, CXCL10-GORASP1, CXCL10-TNFAIP6, DHX58-GEMIN4, DHX58-GORASP1, DHX58-RTP4, DHX58-SAMD9, DHX58-SAMHD1, and DHX58-SIGLEC1. The *x*-axis denotes the time (time: h).

To explore the biological link between arbitrary two proteins, the analysis for the indicator TPC is implemented on these identified DNBs according to the coefficient of variation CV using the gene expression datasets, respectively.  Figure 3 shows the change of the indicator TPC of a small part of the protein pairs from the DNBs from Figure 1 for the disease H3N2. The TPC of other protein pairs for H3N2 is presented in Supplementary Information B. It is obvious that the identified DNBs form a strongly correlated subnetwork to provide the significant warning signal near the critical state (45 h).

From Figures 1–3, it is easy to find that during the predisease state, for DNBs, there are significant differences between the members of the symptomatic group and those of the asymptomatic group which behave in a considerably different manner in terms of three indicators CV, TPD, and TPC that are theoretically derived. However, after the system is driven into the disease state, interestingly the members of both the symptomatic group and the asymptomatic group appear to behave in a manner similar to each other (see the curve after the time *t* = 45 h). Therefore, we can conclude that during the normal state and the disease state, for each protein, the stochastic behaviors for the symptomatic group and the asymptomatic group display similarity. Then, the significant differences for the stochastic behaviors for the symptomatic and asymptomatic groups of DNBs only occur during the predisease state. From this statement, we know that the stochastic signals, such as the indicators CV, TPD, and TPC, make sense for predicting the impending critical transition.

### 3.3. The Criteria to Predict DNBs in Real Datasets for Complex Diseases

Through theoretical analysis presented in Corollaries 2, 4, and 6 in Section 3.1, we obtain three statistical indicators CV, TPC, and TPD that can distinguish the biomarkers from nonbiomarkers. To validate the theoretical results, we calculate these three indicators for three real datasets. Figures 1–3 demonstrate that these three indicators can clearly distinguish the biomarkers and nonbiomarkers when they reach the predisease state. These results can help us to detect the early-warning signals for complex diseases. From Figures 1–3, we can also observe that three indicators exhibit different behaviors after critical transition. That is, the values of the indicators CV and TPC only display a peak at the critical transition point (see *t* = 45 h) while the value of the indicator TPD keeps a high value after the critical transition compared to the value before the critical transition (see *t* = 45 h). We can give their explicit explanations intuitively from the perspectives of the qualitative theory of ordinary differential equations and statistics. First, far before *t* = 45 h, the system is in the normal state or in an asymptomatically stable state from the perspective of the ordinary differential equation. That is, there exist only stable manifolds, or the state has strong attraction or strong robustness. In statistics, this corresponds to the phenomenon that the system fluctuates weakly. Thus, in this state, the fluctuation is weak. Just far after *t* = 45 h, the system is in the disease state or in another asymptomatically stable state. Due to the same reason, in this state, the fluctuation is weak. However, at the bifurcation point, the center manifold occurs. Thus, near *t* = 45 h or in the predisease state, compared with the normal state or the disease state, the system is in a less stable state and has weaker attraction? Thus, in this state, the fluctuation becomes stronger (before *t* = 45 h) or becomes weaker (after *t* = 45 h). In addition, this kind of phenomenon is verified by many real experiments.

Second, in fact, the indicator TPC(*X*, *Y*) = −(1 − |PCC(*X*, *Y*)|) describes the linear correlation relationship between two random variables *X* and *Y* or the similarity relationship between two random variables. Far before *t* = 45 h, the system is in the normal state or in an asymptomatically stable state. In this state, each element of the state variable has different recovery rate, and thus the similarity of their behaviors is weak. Correspondingly, in statistics, the linear correlation relationship is weak. Similarly, we can understand the case in the disease state or after *t* = 45 h. However, near *t* = 45 h or in the predisease state, the dominant eigenvalue is the largest eigenvalue for the Jacobian matrix and often is unique. As we know, the biomarkers are related to the dominant eigenvalue, and their recovery rate can be described by the dominant eigenvalue. Thus, the behaviors of the biomarkers are similar. Thus, in this state, the linear correlation relationship becomes stronger and stronger (before *t* = 45 h) or becomes weaker (after *t* = 45 h). In addition, this kind of phenomenon is verified by many real experiments.

Third, as mentioned above, we know that

(21) $$\begin{array}{c} {TPD}_{i}\left(\;\left[\;a,b\;\right]\;\right)=-\ln\left(\;\overset{b}{\underset{a}{\int }}p_{i}\left(\;s\;\right)ds\;\right), \end{array}$$

where *p*_*i*_(·) is the probability density of the random variable *z*_*i*_(*t*) and *a*, *b* can be taken as the maximum and minimum values of the real data at the initial time instant. We know that the critical transition can be understood as the fact that the system state abruptly switches from one asymptotically stable equilibrium to another one at a critical threshold as the complex disease evolves. In general, we can think that the asymptotically stable equilibrium corresponds to a probability distribution in statistics. Far before *t* = 45 h, the system state is stable keeping the value between the maximum value and the minimum value due to the weak fluctuations, and thus the indicator displays little change. As the critical transition occurs (before *t* = 45 h), the values of some of the proteins gradually evolve beyond the interval [*a*, *b*] due to the stronger and stronger fluctuations, and then the indicator TPD becomes larger and larger. After *t* = 45 h or in the disease state, the values of the biomarkers are all not in the interval [*a*, *b*]. That is, in this state, the system state is another asymptotically stable state. Because this attractor is asymptotically stable, the values of biomarkers cannot return to the interval [*a*, *b*]. Thus, after [*a*, *b*], the indicator TPD displays little change and always keeps a high value.

### 3.4. Functional Analysis of DNBs

To verify the biological significance of the DNBs identified by our method, DAVID was used to carry out functional enrichment analysis, respectively, for the three diseases [27]. The results of function annotation for three datasets showed that the DNBs were significantly enriched in diseases-related biological processes. In H3N2 infection, some DNBs are involved in defense response (e.g., SIGLEC1, TNFAIP6, IRF7, SAMHD1, TLR7, DHX58, and CXCL10) and inflammatory response (e.g., SIGLEC1, TNFAiP6, IRF7, TLR7, and CXCL10). In H1N1 infection, 4 DNBs (SEPT4, NPAT, STMN1, and CCNA1) participate in cell cycle. In the lung injury study, we found that 2 DNBs (Erge and Aplp2) are associated with the regulation of epidermal growth factor receptor activity and epidermal growth factor receptor signaling pathway. At the pathway enrichment level, some pathways were also highly related to complex diseases. For example, for H3N2 influenza, 5 of 22 DNBs (IRF7, TREX1, CASP1, ZBP1, and CXCL10) are observed in the cytosolic DNA-sensing pathway, which plays an important role in initiating innate immunity and adaptive immunity [28]. In addition, another two significantly enriched pathways include RIG-I-like receptor signaling pathway with 3 DNBs (IRF7, DHX58, and CXCL10) and Toll-like receptor signaling pathway with 3 DNBs (IRF7, TLR7, and CXCL10). For H1N1 influenza, 2 DNBs (IRF7, POLR1C) are also observed in the cytosolic DNA-sensing pathway. For acute lung injury, 7 DNBs (Psmb5, Psma1, Psmd13, Psmc3, Psmd4, Psme4, and Psma7) are observed in the pathways of regulating by proteasome mediated degradation, which played a key role in regulating many processes of cellular biology [29, 30].

## 4. Discussion and Conclusions

To detect the predisease state of complex diseases or identify DNBs, we analyzed a series of statistic indicators, such as CV, TPC, and TPD. Although the previous works relevant to DNBs have proposed how to detect the predisease state of complex diseases by using the statistical methods [8, 22, 31], the rigorous mathematical derivation is not given and the networks based on real data are needed to be constructed. In our viewpoints, we improved the mathematical approaches by using a continuous-time dynamical system. Based on a general dynamical model, we theoretically derive the statistical indicators to detect specific early-warning signals for the predisease state to complex diseases. Furthermore, we have also conducted numerical experiments to identify the DNBs based on high-throughput data for three complex diseases. The function analysis verified the meaningfulness of the detected DNBs. In our paper, some statistical indicators, such as skewness and kurtosis, are not theoretically analyzed [10, 14]. It does not mean that the skewness and kurtosis are not statistical characteristics which are useful for us to detect early-warning signals. The analysis whether they can be used as indicators would be important topic in the future.

## Supplementary Material

The supplementary material file includes three parts. The first part A is the derivation of theoretical results for three indicators including CV, TPC, and TPD. The second part B is the numerical simulations for a dynamical network biomarker. The third part C is the Supplementary Figures, including 17 figures.

## Figures and Tables

**Table 1:** DNBs for H3N2, H1N1, and lung with respect to the indicators CV, TPC, and TPD.

Data for diseases	Number for CV	Number for TPD	DNBs
H3N2	264	116	22
			APOL6, CASP1, CNP, CXCL10, DHX58, DRAP1,
			DYNLT1, FAM46A, GEMIN4, GORASP1, IFITM1, IRF7,
			PLSCR1, RTP4, SAMD9, SAMHD1, SIGLEC1, TAF1C,
			TLR7, TNFAIP6, TREX1, ZBP1

H1N1	32	6	22
			ACP6, BTG1, CCNA1, DDX18, DKC1, H2AFV,
			HMGN1, IRF7, ITK, LAX1, NPAT, NR2C1,
			NUCB1, PFN2, POLR1C, RBM4B, RPS2, SEPT4,
			SLBP, SP110, STMN1, VPRBP

Lung	34	104	60
			Abcd3, Actn1, Adcy8, Adss, Anxa1, Aplp2,
			Aqp1, Atp6v1d, Capn1, Clstn1, Csf1r, Dapk1,
			Ddx39, Ensmusg00000050347, Ereg, Faf1, Fzd2, G6pd2,
			Gimap4, Glrx, Gnb1, Gp49a, Grem2, Gtf2i,
			H1f0, Hhip, Hist2h2bb, Hnrnpd, Hprt1, Htra1,
			Kcnq1, Klhl13, Lox, Lrg1, Macf1, Mcee,
			Mmp19, Nagk, Ncl, Nr2f6, Nrp1, Phlpp,
			Pla2g15, Prelp, Prpf40a, Psma1, Psma7, Psmb5,
			Psmc3, Psmd13, Psmd4, Psme4, Rad17, Rad23b,
			Sin3b, Stxbp1, Thbs3, Tjp2, Ulk2, Wbp1
