# Supplementary material for: Several Indicators of Critical Transitions for Complex Diseases Based on Stochastic Analysis
Source: Comput Math Methods Med. 2017 Aug 1;2017:7560758. doi: 10.1155/2017/7560758 (PMC5556999; doi:10.1155/2017/7560758)
Supplement: Supplementary file 1 — The supplementary material file includes three parts. The first part A is the derivation of theoretical results for three indicators including CV, TPC, and TPD. The second part B is the numerical simulations for a dynamical network biomarker. The third part C is the Supplementary Figures, including 17 figures. [file 7560758.f1.pdf]

Supplementary Information:
Several indicators of critical transitions for complex biological systems based on stochastic analysis

Gang Wang, Yuanyuan Li, Xiufen Zou

Contents

A. Derivation of theoretical results for three indicators . . . . . …………….. . . … . . .. . . …... **S2**
A1 A generic model in abstract phase space and some preliminaries. . . . . ….. . … S2
A2 Stochastic analyses and quantitative indicators. . . . .…. . . …. .S6
A2.1 Coefficient of Variation (CV). . . . .…. . S8
A2.2 Transformed Pearson's correlation coefficient (TPC). . . . . … .S10
A2.3 Transformed Probability distribution (TPD) .. . .………... ... .S12
A2.4 Summary. S14

B. Numerical simulations for a dynamical network biomarker. **S14**

C. Supplementary Figures. . . . . …S17
C1. The dataset for H3N2. . . . . S17
C2. The dataset for H1N1. . . . .S20
C3. The dataset for Lung. . . . . … S21

A. Derivation of theoretical results for three indicators

A1. A generic model in abstract phase space and some preliminaries

Consider the following continuous-time dynamical system representing the dynamical evolution of a complex system:

$$\frac{dZ(t)}{dt} = f\left(Z(t); P\right), \tag{S1}$$

where $Z(t) = \left(z_1(t), z_2(t), \cdots, z_n(t)\right)^T \in \mathbb{R}^n$ is an n-dimensional state vector or variables at time instant t representing gene or protein expressions, and $P = \left(p_1, p_2, \cdots, p_s\right)^T \in \mathbb{R}^s$ is an s-dimensional parameter vector or driving factors representing slowly changing factors. $f : \mathbb{R}^n \times \mathbb{R}^s \to \mathbb{R}^n$ an n-dimensional nonlinear function vector.

For the convenience for our derivations in next section, several theoretical conclusion should be listed here.

Definition A1.1(Kuznetsov, 2013) A dynamical system $\left\{T, \mathbb{R}^n, \varphi^t\right\}$ is called topologically equivalent to a dynamical system $\left\{T, \mathbb{R}^n, \psi^t\right\}$ where φ^t or ψ^t is a flow for the corresponding continuous-time model if there is a homeomorphism $h : \mathbb{R}^n \to \mathbb{R}^n$ mapping orbits of the first system onto orbits of the second system, preserving the direction of time.

As the parameters vary, the phase portrait also varies. There are two possibilities: either the system remains topologically equivalent to the original one, or its topology changes.

Definition A1.2(Kuznetsov, 2013**)** The appearance of a topologically nonequivalent phase portrait under variation of parameters P is called a bifurcation.

Thus, a bifurcation is a change of the topological type of the system as its parameters pass through a bifurcation value P^*.

Theorem A1.1(Kuznetsov, 2013**)** Suppose that Z_0 is an equilibrium of (S1), and denote by A the Jacobian matrix of $f\left(x\right)$ evaluated at the equilibrium, i.e. $A = f_Z\left(Z_0\right)$. Then Z_0 is asymptotically stable if all eigenvalues $\lambda_1, \lambda_2, \cdots, \lambda_n$ of A satisfy $\operatorname{Re}\lambda < 0$.

Theorem A1.2(Kuehn, 2013**)**Suppose $F = \left(Z^*, P^*\right)$ is Lyapunov stable with respect to the fast subsystem

$$\begin{cases} \dfrac{dZ}{dt} = f\left(Z, P\right) \\ \dfrac{dP}{dt} = 0 \end{cases}, \tag{S2}$$

then there is no critical transition at F.

We can also view this theorem as the robustness for the asymptotically stable equilibrium Z^* with respect to the parameter P^* (Hart, et al., 2012).

Obviously, our focus with respect to complex diseases is critical transitions. And it is also appropriate to use the saddle-node bifurcation, which is a kind of critical transition(Kuehn, 2011; Kuehn, 2013), to depict the evolution from normal state to disease state, because the deterioration for complex diseases is just the situation that an asymptotically stable equilibrium "fall" into another asymptotically equilibrium abruptly. We can view the bifurcation point as transition point in this paper. According to Theorem A2.1 and A2.2, the bifurcation point considered here is not hyperbolic. That is, for the Jacobian matrix of the continuous-time dynamical system at the bifurcation point, the real part of one of the eigenvalues is zero.

Based on this conclusion, assume that the following conditions hold for (S1), for which the bifurcation occurs at the bifurcation value P^*.

1°. Z^* is an equilibrium of system (S1)such that $f\left(Z^*;P\right)=0$.

2°. For the bifurcation value P^*, the real parts of one or a complex-conjugate pair of the eigenvalues for the Jacobian matrix $\left.\frac{\partial f\left(Z;P^*\right)}{\partial Z}\right|_{Z=Z^*}$ are equal to zero.

3°.When $P \neq P^*$, the real parts of all the eigenvalues for the Jacobian matrix are negative.

Without loss of generality, let $X\left(t\right)=Z\left(t\right)-Z^*$ which implies that the equilibrium Z^* corresponds to $X^*=0$. Thus, in the vicinity of $X^*=0$, system (S1)is transformed into

$$\frac{dX\left(t\right)}{dt}=f\left(X\left(t\right)+Z^*;P\right), \tag{S3}$$

where $X\left(t\right)=\left(x_1\left(t\right),x_2\left(t\right),\cdots,x_n\left(t\right)\right)^n\in\mathbb{R}^n$. We can write

$$J=\left.\frac{\partial f\left(Z;P\right)}{\partial Z}\right|_{Z=Z^*}$$

as the Jacobian matrix of f, and $N\left(X\left(t\right);P\right)$ as the nonlinear part containing higher-order terms. Then, taking the Taylor expansion for f at the equilibrium $X^*\equiv 0$ gives

$$\frac{dX\left(t\right)}{dt}=JX\left(t\right)+N\left(X\left(t\right);P\right), \tag{S4}$$

where $J=J\left(P\right)$ is nonsingular. It is easy to see that, there exists a nonsingular matrix $S\triangleq S\left(P\right)$ satisfying $J=S\Lambda S^{-1}$, where Λ is in a normalized form. If we make the transformation $Y\left(t\right)=S^{-1}X\left(t\right)$ and take the derivative with respect to the time t on both sides, a linearized equation of (S4) with the random perturbations can be formulated as(Mao, 2007)

$$\frac{dY\left(t\right)}{dt}=\Lambda Y\left(t\right)+G\frac{dB\left(t\right)}{dt}, \tag{S5}$$

or

$$\begin{pmatrix} dy_1(t) \\ dy_2(t) \\ \vdots \\ dy_n(t) \end{pmatrix} = \Lambda \begin{pmatrix} y_1(t) \\ y_2(t) \\ \vdots \\ y_n(t) \end{pmatrix} + \begin{pmatrix} g_{11} & g_{12} & \cdots & g_{1m} \\ g_{21} & g_{22} & \cdots & g_{2m} \\ \vdots & \vdots & & \vdots \\ g_{n1} & g_{n2} & \cdots & g_{nm} \end{pmatrix} \begin{pmatrix} dB_1(t) \\ dB_2(t) \\ \vdots \\ dB_m(t) \end{pmatrix}, \tag{S6}$$

where $Y(t) = (y_1(t), y_2(t), \cdots, y_n(t))^T$, and $\Lambda = \Lambda(P)$. $B(t) = (B_1(t), \cdots, B_m(t))^T$ is an m-dimensional Brownian motion and

$$G = \begin{pmatrix} g_{11} & g_{12} & \cdots & g_{1m} \\ g_{21} & g_{22} & \cdots & g_{2m} \\ \vdots & \vdots & & \vdots \\ g_{n1} & g_{n2} & \cdots & g_{nm} \end{pmatrix} \in \mathbb{R}^{n\times m}.$$

In order to make the stochastic differential model (S6)meaningful, we can assume that

$$\sum_{l=1}^{m} g_{il} g_{jl} \neq 0, i, j = 1, 2, \cdots, n. \tag{S7}$$

Notice that J and Λ have the same eigenvalues, and thus the conditions 2° and 3° hold for Λ. Therefore, it is reasonable for us to call the eigenvalue with the largest real part the dominant eigenvalue, the real part of which first reaches 0 when P reaches P^*.

Obviously, the normalized form Λ for the Jacobian matrix J is different for different nonlinear function f in (S3). Before P reaches the bifurcation value P^*, the eigenvalues for J or Λ can be real or complex. Therefore, we can consider the following three cases.

Case 1. If J have n real eigenvalues and n eigenvectors, then there exists a singular matrix S satisfying $\Lambda = S^{-1}JS = diag(\lambda_1, \lambda_2, \cdots, \lambda_n)$, where $\lambda_i < 0 (i = 1, 2, \cdots, n)$. In this case, we can view λ_1 as the dominant eigenvalue.

Case 2. If J have n real eigenvalues but does not have n linearly independent eigenvectors, there is a nonsingular matrix S making

$$\Lambda = S^{-1}JS = \begin{pmatrix} G_1 & 0 & \cdots & 0 \\ 0 & \ddots & \ddots & \vdots \\ \vdots & \ddots & \ddots & 0 \\ 0 & \cdots & 0 & G_q \end{pmatrix}, \tag{S8}$$

where

$$G_j = \begin{pmatrix} \lambda_j & 1 & 0 & \cdots & 0 \\ 0 & \ddots & \ddots & \ddots & \vdots \\ \vdots & \ddots & \ddots & \ddots & 0 \\ \vdots & & \ddots & \ddots & 1 \\ 0 & \cdots & \cdots & 0 & \lambda_j \end{pmatrix},$$

with $\lambda_j < 0$. We can move the block with the dominant eigenvalue to the first position of Jordan normal form Λ.

Case 3 . If J has at least one pair of complex conjugate eigenvalues, then there is a nonsingular matrix S making $\Lambda = S^{-1}JS$ where the partitioned matrix Λ is of the form (S8), but

$$G_j = \begin{pmatrix} C_j & I & 0 & \cdots & 0 \\ 0 & \ddots & \ddots & \ddots & \vdots \\ \vdots & \ddots & \ddots & \ddots & 0 \\ \vdots & & \ddots & \ddots & I \\ 0 & \cdots & \cdots & 0 & C_j \end{pmatrix}, \quad C_j = \begin{pmatrix} a_j & -b_j \\ b_j & a_j \end{pmatrix}, I = \begin{pmatrix} 1 & \\ & 1 \end{pmatrix},$$

where $a_j < 0, b_j \in \mathbb{R}$ before P reaches the bifurcation value P^*. Also suppose that the dominant eigenvalues are in the first position of Λ.

A2. Stochastic analyses and statistical indicators

In Case 1, since

$$\Lambda = \begin{pmatrix} \lambda_1 & & & \\ & \lambda_2 & & \\ & & \ddots & \\ & & & \lambda_n \end{pmatrix}, \lambda_j < \lambda_1 < 0, j = 2, 3, \cdots, n,$$

we have

$$y_i(t) = e^{\lambda_i (t-t_0)} y_i(t_0) + \sum_{k=1}^{m} g_{ik} \int_{t_0}^{t} e^{\lambda_i (t-s)} dB_k(s), i = 1, 2, \cdots, n. \tag{S9}$$

Then,

$$Var(y_i(t)) = \left(\sum_{k=1}^{m} g_{ik}^{\ 2} \right) \frac{1 - e^{2\lambda_i (t-t_0)}}{-2\lambda_i}. \tag{S10}$$

In Case 2, based on the similarity of the methods, set $j = 2$ as the order of the G_1 block. Solving the stochastic differential equations

$$\begin{pmatrix} dy_1(t) \\ dy_2(t) \end{pmatrix} = \begin{pmatrix} \lambda_1 & 1 \\ & \lambda_1 \end{pmatrix} \begin{pmatrix} y_1(t) \\ y_2(t) \end{pmatrix} dt + \sum_{k=1}^{m} \begin{pmatrix} g_{1k} \\ g_{2k} \end{pmatrix} dB_k(t) \tag{S11}$$

yields

$$\begin{cases} y_1(t) = e^{\lambda_1 (t-t_0)} [y_1(t_0) + (t - t_0) y_2(t_0)] \\ \qquad\qquad + \sum_{k=1}^{m} \int_{t_0}^{t} e^{\lambda_1 (t-s)} [g_{1k} + (t-s) g_{2k}] dB_k(s) \\ y_2(t) = e^{\lambda_1 (t-t_0)} y_2(t_0) + \sum_{k=1}^{m} \int_{t_0}^{t} g_{2k} e^{\lambda_1 (t-s)} dB_k(s) \end{cases}. \tag{S12}$$

It is easy to see that

$$Var(y_1(t))=\left(\sum_{k=1}^{m} g_{1k}{}^2\right)\frac{1-e^{2\lambda_1(t-t_0)}}{-2\lambda_1} + \left(\sum_{k=1}^{m} g_{1k}g_{2k}\right)\frac{[2\lambda_1(t-t_0)-1]e^{2\lambda_1(t-t_0)}+1}{2\lambda_1{}^2} + \left(\sum_{k=1}^{m} g_{2k}{}^2\right)\frac{[2\lambda_1{}^2(t-t_0)^2-2\lambda_1(t-t_0)+1]e^{2\lambda_1(t-t_0)}-1}{4\lambda_1{}^3} \quad . \tag{S13}$$

and $Var\left(y_2\left(t\right)\right)$ is the same as (S10).

In case 3, similar to Case 2, suppose $j=2$ is the order of the G_1 block. Solving

$$\begin{pmatrix} dy_1(t) \\ dy_2(t) \end{pmatrix}=\begin{pmatrix} a_1 & -b_1 \\ b_1 & a_1 \end{pmatrix}\begin{pmatrix} y_1(t) \\ y_2(t) \end{pmatrix}dt+\sum_{k=1}^{m}\begin{pmatrix} g_{1k} \\ g_{2k} \end{pmatrix}dB_k(t) \tag{S14}$$

yields

$$\begin{cases} y_1(t)=e^{a_1(t-t_0)}(\cos(b_1(t-t_0))y_1(t_0)-\sin(b_1(t-t_0))y_2(t_0)) \\ \qquad +\sum_{k=1}^{m}\int_{t_0}^{t} e^{a_1(t-s)}[\cos(b_1(t-s))g_{1k}-\sin(b_1(t-s))g_{2k}]dB_k(s) \\ y_2(t)=e^{a_1(t-t_0)}(\sin(b_1(t-t_0))y_1(t_0)+\cos(b_1(t-t_0))y_2(t_0)) \\ \qquad +\sum_{k=1}^{m}\int_{t_0}^{t} e^{a_1(t-s)}[\sin(b_1(t-s))g_{1k}+\cos(b_1(t-s))g_{2k}]dB_k(s) \end{cases} . \tag{S15}$$

Based on this, it is easy to verify that

$$\begin{cases} Var\left(y_1\left(t\right)\right)=\frac{\sum_{k=1}^{m} g_{1k}{}^2-\sum_{k=1}^{m} g_{2k}{}^2}{2}I_{\cos}-\left(\sum_{k=1}^{m} g_{1k}g_{2k}\right)I_{\sin} \\ \qquad +\frac{\sum_{k=1}^{m} g_{1k}{}^2+\sum_{k=1}^{m} g_{2k}{}^2}{-4a_1}\left(1-e^{2a_1(t-t_0)}\right) \\ Var\left(y_2\left(t\right)\right)=\frac{\sum_{k=1}^{m} g_{2k}{}^2-\sum_{k=1}^{m} g_{1k}{}^2}{2}I_{\cos}+\left(\sum_{k=1}^{m} g_{1k}g_{2k}\right)I_{\sin} \\ \qquad +\frac{\sum_{k=1}^{m} g_{1k}{}^2+\sum_{k=1}^{m} g_{2k}{}^2}{-4a_1}\left(1-e^{2a_1(t-t_0)}\right) \end{cases} , \tag{S16}$$

where

$$\begin{cases} I_{\sin} = \dfrac{\left[a_1\sin(2b_1(t-t_0)) - b_1\cos(2b_1(t-t_0))\right]e^{2a_1(t-t_0)} + b_1}{2(a_1^{\ 2}+b_1^{\ 2})} \\ I_{\cos} = \dfrac{[b_1\sin(2b_1(t-t_0)) + a_1\cos(2b_1(t-t_0))]e^{2a_1(t-t_0)} - a_1}{2(a_1^{\ 2}+b_1^{\ 2})} \end{cases}.$$

In view of the above three cases, we can get

$$\lim_{t\to+\infty}\lim_{P\to P^*} Var\left(y_i\left(t\right)\right) = \begin{cases} +\infty & i=1 \\ finite\ number & i\neq 1 \end{cases}. \tag{S17}$$

We will investigate statistical characteristics for the original variables Z. Here we should first point out the fact that: from $X\left(t\right)=Z\left(t\right)-Z^*$ and $Y\left(t\right)=S^{-1}X\left(t\right)$, it follows that

$$Z(t)=SY(t)+Z^*, \tag{S18}$$

or, equivalently,

$$z_i(t)=\sum_{j=1}^{n} s_{ij}y_j(t)+z_i^*, i=1,2,\cdots,n, \tag{S19}$$

where $S \triangleq \left(s_{ij}\right)_{n\times n}$.

A2.1. Coefficient of Variation (CV)

According to the definition of coefficient of variation $CV\left(X\right)=\dfrac{\sqrt{Var\left(X\right)}}{E\left(X\right)}$,

we can consider Case 1 for which Λ is diagonal and it is easy for us to calculate $Var\left(X\right)$ and $E\left(X\right)$.

Theorem 3.1.1 Suppose that Λ Eq. (3) in main text, i.e., Eq.(S5)in Supplementary information, is diagonal, then

$$\lim_{t\to+\infty}\lim_{P\to P^*} CV\left(z_i\left(t\right)\right) = \begin{cases} +\infty & s_{i1}\neq 0 \\ \text{A}\ \ finite\ number & s_{i1}=0 \end{cases}. \tag{S20}$$

Proof. If $i\neq j$, then

$$\begin{aligned} Cov(y_i(t),y_j(t)) &= E\left[\left(\sum_{k=1}^{m}\int_{t_0}^{t} e^{\lambda_i(t-s)}g_{ik}dB_k(s)\right)\left(\sum_{k=1}^{m}\int_{t_0}^{t} e^{\lambda_j(t-s)}g_{jk}dB_k(s)\right)\right] \\ &= \left(\sum_{k=1}^{m} g_{ik}g_{jk}\right)\left(\frac{1-e^{(\lambda_i+\lambda_j)(t-t_0)}}{-(\lambda_i+\lambda_j)}\right) \end{aligned}.$$

Thus,

$$\lim_{t\to+\infty}\lim_{P\to P^*} Cov\left(y_i\left(t\right),y_j\left(t\right)\right) = \frac{\sum_{k=1}^{m} g_{ik}g_{jk}}{-\left(\lambda_i+\lambda_j\right)}. \tag{S21}$$

From (S19), we have

$$z_i(t) = s_{i1}y_1(t) + \cdots + s_{in}y_n(t) + z_i^*$$

and

$$Var(z_i(t)) = s_{i1}{}^2 Var(y_1(t)) + \sum_{l=2}^{n} s_{il}{}^2 Var(y_l(t)) + \sum_{k=1}^{n}\sum_{\substack{m=1\\ m\neq k}}^{n} s_{ik}s_{im}Cov\left(y_k(t), y_m(t)\right).$$

Then,

$$\lim_{t\to+\infty}\lim_{P\to P^*} Var\left(z_i(t)\right) = \begin{cases} +\infty & s_{i1}\neq 0 \\ \text{A } \textit{finite number} & s_{i1} = 0 \end{cases}.$$

Since

$$\lim_{t\to+\infty}\lim_{P\to P^*} E\left(y_i(t)\right) = \begin{cases} y_1(t_0) & i = 1 \\ 0 & i \neq 1 \end{cases},$$

we know that

$$\lim_{t\to+\infty}\lim_{P\to P^*} E\left(z_i(t)\right) = \begin{cases} s_{i1}y_1(t_0) + z_i^* & s_{i1}\neq 0 \\ z_i^* & s_{i1} = 0 \end{cases}.$$

According to $CV(X) = \frac{\sqrt{Var(X)}}{E(X)}$, it is easy to provethat

$$\lim_{t\to+\infty}\lim_{P\to P^*} CV\left(z_i(t)\right) = \begin{cases} +\infty & s_{i1}\neq 0 \\ \text{A } \textit{finite number} & s_{i1} = 0 \end{cases}.$$

This completes the proof.

We recall that $z_i(t) = s_{i1}y_1(t) + \cdots + s_{in}y_n(t) + z_i^*$. $s_{i_1 1}\neq 0$ implies that $z_{i_1}(t)$ is related to the dominant eigenvalue. $s_{i_1 1}\neq 0$ and $s_{i_2 1} = 0$ mean that $z_{i_1}(t) = \sum_{l=1}^{n} s_{i_1 l}y_l(t) + z_{i_1}^*$ may lead to the critical transition and $z_{i_2}(t) = \sum_{l=2}^{n} s_{i_2 l}y_l(t) + z_{i_2}^*$ may not lead to the critical transition. For the convenience to discuss in the following sections, we denote $z_{i_1}(t)$ and $z_{i_2}(t)$ as a biomarker and a non-biomarker, respectively.

From above discussion, we prove that three following descriptions are equivalent.

(a) The dominant eigenvalue of dynamical system tends to 0（$\lambda_1 \to 0$）

(b) The parameter P approaches to the bifurcation value P^* $(P \to P^*)$

(c) The system is close to the critical transition

Then, we can prove the following corollary.

Corollary 3.1.1 As the parameter P approaches the bifurcation value P^*, i.e, the system is close to the critical transition,

(1) There are no drastic changes for the coefficient of variation CV of non-biomarkers.

(2) The coefficient of variation CV for biomarkers is much larger than the coefficient of variation CV for non-biomarkers.

(3) The coefficient of variation CV for biomarkers drastically increases,

Proof.(1)According to Theorem 3.1.1, we know that the limit for non-biomarker $\lim_{t\to+\infty}\lim_{P\to P^*} CV\left(z_{i_2}(t)\right)$ is finite, which means that there are no drastic changes for the coefficient of variation of non-biomarkers.

(2) It is easy to see that

$$\lim_{t\to+\infty}\lim_{P\to P^*}\frac{CV\left(z_{i_1}(t)\right)}{CV\left(z_{i_2}(t)\right)}=+\infty.$$

Thus, for arbitrary $G_1>1$, there exists $T_1>0$ such that when $t>T_1$, it follows that

$$\lim_{P\to P^*}\frac{CV\left(z_{i_1}(t)\right)}{CV\left(z_{i_2}(t)\right)}>G_1.$$

Further, there exists $\delta_1=\delta_1(t)>0$ such that $P\in\left(P^*-\delta_1,P^*\right)$, it follows that

$$\frac{CV\left(z_{i_1}(t)\right)}{CV\left(z_{i_2}(t)\right)}>G_1,$$

which implies that the conclusion (2) is valid.

(3) For sufficiently small $\delta_1^*>0$ and $P_0\in\left(P^*-\delta_1^*,P^*\right)$, it is easy to see that $\lim_{t\to+\infty} CV\left(z_{i_1}(t,P_0)\right)$ exists, and

$$\lim_{t\to+\infty}\lim_{P\to P^*}\frac{CV\left(z_{i_1}(t,P)\right)}{CV\left(z_{i_1}(t,P_0)\right)}=+\infty.$$

Then for arbitrary $G_2>1$, there exists $T_2>0$ such that when $t>T_2$, it follows that

$$\lim_{P\to P^*}\frac{CV\left(z_{i_1}(t,P)\right)}{CV\left(z_{i_1}(t,P_0)\right)}>G.$$

Thus, there exists $\delta_2=\delta_2(t)>0$ such that $P\in\left(P^*-\delta_2,P^*\right)$, it follows that

$$\frac{CV\left(z_{i_1}(t,P)\right)}{CV\left(z_{i_1}(t,P_0)\right)}>G,$$

which implies that Corollary 3.1 (3) is valid.

A2.2.Transformed Pearson's correlation coefficient (TPC)

As we know, $-1\le PCC(X,Y)\le 1$, where $PCC(X,Y)$ is Pearson's correlation coefficient between the random variables X and Y. To make it more effective in practice, we can define an indicator with respect to Pearson's correlation coefficient as follows

$$TPC(X,Y)=-\ln\left(1-\left|PCC(X,Y)\right|\right). \qquad \text{(S22)}$$

Theorem 3.1.2 Suppose that Λ in Eq.(S5) is diagonal, then

$$\lim_{t\to+\infty}\lim_{P\to P^*} TPC\left(z_i(t),z_j(t)\right)=\begin{cases}+\infty & s_{i1}\ne 0, s_{j1}\ne 0\\ 0 & s_{i1}=0, s_{j1}\ne 0.\\ \text{A } \textit{finite number} & s_{i1}=0, s_{j1}=0\end{cases} \qquad \text{(S23)}$$

Proof. We can easily obtain that

$$Cov(z_i(t), z_j(t)) = E[(\sum_{k=1}^{n} s_{ik}(y_k(t) - Ey_k(t)))(\sum_{m=1}^{n} s_{jm}(y_m(t) - Ey_m(t)))]$$
$$= s_{i1}s_{j1}Var(y_1(t)) + \cdots + s_{in}s_{jn}Var(y_n(t))$$
$$+ \sum_{k=1}^{n}\sum_{\substack{m=1 \\ m\neq k}}^{n} s_{ik}s_{jm}Cov\left(y_k(t), y_m(t)\right).$$

Specifically, it is easy to see that

$$Var\left(z_i(t), z_i(t)\right) = s_{i1}{}^2 Var\left(y_1(t)\right) + \sum_{l=2}^{n} s_{il}{}^2 Var\left(y_n(t)\right)$$
$$+ \sum_{k=1}^{n}\sum_{\substack{m=1 \\ m\neq k}}^{n} s_{ik}s_{im}Cov\left(y_k(t), y_m(t)\right).$$

For the discussion for Pearson's correlation coefficient, we should suppose $1 \le i \ne j \le n$. From (S17),(S21) and the definition formula for Pearson's correlation coefficient, i.e.,

$$PCC\left(z_i(t), z_j(t)\right) = \frac{Cov\left(z_i(t), z_j(t)\right)}{\sqrt{Var\left(z_i(t)\right)Var\left(z_j(t)\right)}},$$

It follows that

$$\lim_{t\to+\infty}\lim_{P\to P^*}\left|PCC\left(z_i(t), z_j(t)\right)\right| = \begin{cases} 1 & s_{i1} \ne 0, s_{j1} \ne 0 \\ 0 & s_{i1} = 0, s_{j1} \ne 0, \\ \alpha & s_{i1} = 0, s_{j1} = 0 \end{cases}$$

where $\alpha \in (0,1)$. Thus, (S23) is valid.

This completes the proof.

Corollary 3.1.2 As the parameter P approaches the bifurcation value P^*, i.e, the system is close to the critical transition,

(1)There are no drastic changes for the indicator TPC between non-biomarkers.

(2)The indicator TPC between biomarkers is much larger than TPC between non-biomarkers.

(3)The indicator TPC between a biomarker and a non-biomarker is much smaller than TPC between non-biomarkers.

(4) The indicator TPC between biomarkers drastically increases.

Proof. (1)According to Theorem 3.1.2, we know that the limit of the indicator TPC between non-biomarkers is non-zero and finite, which implies the following corollary validates.

For convenience of the following discussion, suppose that Λ in Eq.(S5) is diagonal, and $s_{i_1 1} \ne 0, s_{i_2 1} \ne 0, s_{i_3 1} = 0, s_{i_4 1} = 0$, which means that $z_{i_1}(t), z_{i_2}(t)$ are biomarkers and $z_{i_3}(t), z_{i_4}(t)$ are non-biomarkers based on the definition $z_i(t) = \sum_{l=1}^{n} s_{il}y_l(t) + z_i^*$.

(2) According to Theorem 3.1.2, we obtain

$$\lim_{t\to+\infty}\lim_{P\to P^*}\frac{TPC\left(z_{i_1}(t),z_{i_2}(t)\right)}{TPC\left(z_{i_3}(t),z_{i_4}(t)\right)}=+\infty.$$

Then, for arbitrary $G_3>1$, there exists $T_3>0$ such that when $t>T_3$, it follows that

$$\lim_{P\to P^*}\frac{TPC\left(z_{i_1}(t),z_{i_2}(t)\right)}{TPC\left(z_{i_3}(t),z_{i_4}(t)\right)}>G_3.$$

Furthermore， there exists $\delta_3=\delta_3(t)>0$ such that when $P\in\left(P^*-\delta_3,P^*\right)$,it follows that

$$\frac{TPC\left(z_{i_1}(t),z_{i_2}(t)\right)}{TPC\left(z_{i_3}(t),z_{i_4}(t)\right)}>G_3.$$

(3) Similarly, in view of the fact that

$$\lim_{t\to+\infty}\lim_{P\to P^*}\frac{TPC\left(z_{i_3}(t),z_{i_4}(t)\right)}{TPC\left(z_{i_1}(t),z_{i_3}(t)\right)}=+\infty,$$

we easily see that the indicator $TPC\left(z_{i_1}(t),z_{i_3}(t)\right)$ between a biomarker and a non-biomarker is much smaller than $TPC\left(z_{i_3}(t),z_{i_4}(t)\right)$ between non-biomarkers.

(4) For sufficiently small $\delta_2^*>0$ and $P_0\in\left(P^*-\delta_2^*,P^*\right)$, we can see that $\lim_{t\to+\infty}TPC\left(z_{i_1}(t,P_0),z_{i_2}(t,P_0)\right)$ exists and thus there exists an upper bound $\beta>0$ for $TPC\left(z_{i_1}(t,P_0),z_{i_2}(t,P_0)\right)$.

Since $\lim_{t\to+\infty}\lim_{P\to P^*}TPC\left(z_{i_1}(t),z_{i_2}(t)\right)=+\infty$, then for arbitrary $G_4>0$, there exists $T_4>0$ such that when $t>T_4$, it follows that

$$\lim_{P\to P^*}TPC\left(z_{i_1}(t),z_{i_2}(t)\right)>G_4\beta.$$

Hence, there exists $\delta_4=\delta_4(t)>0$ such that $P\in\left(P^*-\delta_4,P^*\right)$, it follows that

$$TPC\left(z_{i_1}(t,P),z_{i_2}(t,P)\right)>G_4\beta\ge G_4\left(TPC\left(z_{i_1}(t,P_0),z_{i_2}(t,P_0)\right)\right).$$

A2.3.Transformed probability distribution (TPD)

Obviously,

$$z_i(t)\sim N(\mu_i(t),(\sigma_i(t))^2),\quad i=1,2,\cdots,n,$$

where

$$\mu_i(t)=E\left(z_i(t)\right)$$
$$\sigma_i(t)=\sqrt{Var\left(z_i(t)\right)}.$$

Thus, the probability density of the random variable $z_i(t)$ is

$$p_i(x)=\frac{1}{\sqrt{2\pi}\sigma_i}e^{-\frac{(x-\mu_i)^2}{2\sigma_i^2}}. \qquad \text{(S24).}$$

Define

$$TPD_i\left([a,b]\right) = -\ln\left(\int_a^b p_i(s)ds\right). \qquad \text{(S25)}$$

Theorem 3.1.3 Suppose that Λ in Eq.(S5) is diagonal, then

$$\lim_{t\to+\infty}\lim_{P\to P^*} TPD_i\left([a,b]\right) = \begin{cases} +\infty & s_{i1} \neq 0 \\ \text{A } \textit{finite number} & s_{i1} = 0 \end{cases}, \qquad \text{(S26)}$$

where $0 < a < b$.

Proof. It is easy to obtain that

$$\lim_{t\to+\infty}\lim_{P\to P^*} TPD_i\left([a,b]\right) = \lim_{t\to+\infty}\lim_{P\to P^*}\left[-\ln\left(\int_a^b \frac{1}{\sqrt{2\pi}\sigma_i} e^{-\frac{(x-\mu_i)^2}{2\sigma_i^2}} dx\right)\right]$$

$$= \lim_{t\to+\infty}\lim_{P\to P^*}\left[-\ln\left(\frac{1}{\sqrt{2\pi}\sigma_i}\int_{\frac{a-\mu_i}{\sqrt{2}\sigma_i}}^{\frac{b-\mu_i}{\sqrt{2}\sigma_i}} e^{-u^2} d\left(\left(\sqrt{2}\sigma_i\right)u+\mu_i\right)\right)\right]$$

$$= \lim_{t\to+\infty}\lim_{P\to P^*}\left[-\ln\left(\frac{1}{\sqrt{\pi}}\int_{\frac{a-\mu_i}{\sqrt{2}\sigma_i}}^{\frac{b-\mu_i}{\sqrt{2}\sigma_i}} e^{-u^2} du\right)\right] .$$

$$-\ln\left(\frac{1}{\sqrt{\pi}}\int_{\lim_{t\to+\infty}\lim_{P\to P^*}-\frac{a-\mu_i}{\sqrt{2}\sigma_i}}^{\lim_{t\to+\infty}\lim_{P\to P^*}\frac{b-\mu_i}{\sqrt{2}\sigma_i}} e^{-u^2} du\right)$$

$$= \begin{cases} +\infty & s_{i1} \neq 0 \\ \text{A } \textit{finite number} & s_{i1} = 0 \end{cases}$$

This completes the proof.

Remark. Note that in practice, we often choose the minimum value for the data at the initial time as a and choose the maximum value for the data at the initial time as b. In this way, it is appropriate to use the indicator TPD to depict the change of the probability distribution as the parameter P approaches P^*.

Similarly, we can easily obtain the corresponding corollary.

Corollary 3.1.3 As the parameter P approaches the bifurcation value P^*, i.e, the system is close to the critical transition,

(1) There are no drastic changes for the probability distribution of non-biomarkers.

(2)The probability distribution for biomarkers is much larger than the probability distribution for non-biomarkers.

(3)The probability distribution for biomarkers drastically increases.□

A2.4. Summary

Based on the above analyses, we know that the statistical properties changes significantly for the variables z_i with $s_{i1} \neq 0$ as the parameter P approaches the bifurcation value P^*, and thus all these variables z_i with $s_{i1} \neq 0$ form a group, called the dominant group, which will first "fall" into the disease state. It is

easy to see that, the dominant group characterizes the dynamical features of the underlying system, and the variables in the group are strongly and dynamically correlated in the pre-disease state. We can find this by noticing that as the parameter P approaches the bifurcation value P^*, the indicator TPC or Pearson's correlation coefficient PCC between any two members among the dominant group increases significantly, and the indicator TPC between each of the dominant group and other variables with $s_{i1} = 0$ decreases significantly. Therefore, these variables in the dominant group form a subnetwork from a network viewpoint. Hence, we also defined it as a DNB.

As the parameter P approaches the bifurcation value P^*, the indicator CV for biomarkers increases greatly, and much larger than that for non-biomarkers, which implies that during this process, the biomarkers fluctuates greatly(Scheffer, et al., 2009; Scheffer, et al., 2015).

Note that the autocorrelation and the probability distribution TPD for biomarkers display similar laws. The change of autocorrelation indicates that as the parameter P approaches the bifurcation value P^*, the linear relationship between the behaviors of one biomarker at two distinct time instants strengthens greatly. And the change of the indicator TPD demonstrates that the probability distribution for biomarkers changes greatly, or the states for biomarkers deviate drastically from the original states as the critical transition occurs.

Here we note that the conditions derived for the variables with $s_{i1} \neq 0$ are in fact the generic properties of the DNB members in dynamics whenever the system approaches a critical tipping point. Such critical behavior is mainly due to the occurrence of the dominant eigenvalue with real part zero(Dai, et al., 2013; Lade and Gross, 2012; Van Nes and Scheffer, 2007; Veraart, et al., 2012), which is relevant to the so-called "critical slowing down" phenomenon (Chisholm and Filotas, 2009; Scheffer, et al., 2009).

In biological systems, the concentrations of molecules in the DNB tend to increasingly fluctuate when the system approaches the critical transition point, and change in a strongly collective manner, which is the key feature of a DNB. Hence, the existence of the DNB indicates that the system is in the pre-disease state.

System (S1) is used to describe the progression of a disease. As the parameter P approaches the critical value P^*, which leads to $\mathrm{Re}(\lambda_i) \to 0$, the system reaches the pre-disease state. In order to investigate the stochastic behavior of the variables, we assume that the real perturbation can be described by the white Gaussian noises, and obtain the stochastic differential equations (S5). Using this stochastic model, we calculate the expressions for each variable. Then, the expression of each statistical indicator is calculated, and we analyze the change of them. To summarize the above analysis, we have several theorems listed previously.

In fact, at any instant of disease progression, the expression of each variable (the concentration of one observable) may stochastically change due to perturbations. Hence in most complex diseases, it is difficult to identify the early-warning signal by focusing on some variables and indicators statically. In our study, our detection of early-warning signals is based on a network of dynamically correlated observables. In view of the theoretical results, we can identify the dominant group or the DNB if the system approaches the pre-disease state.

Mathematical models based on bifurcation theory and center manifold theory have successfully been applied to many fields for describing the catastrophic phenomena near the critical point(Dai, et al., 2015; Dakos, et al., 2010; Drake and Griffen, 2010; Puu, 2013; Scheffer, et al., 2009; Scheffer, et al., 2001). There is no exception for complex diseases (Eikenberry, et al., 2009; Hirata, et al., 2010; Huang, et al., 2009;Tanaka, et al., 2008). Generally, the dynamics for the progression of complex diseases are usually constructed in a high-dimensional space with a large number of variables and parameters. However, provided that the system driven by some (unknown) parameters approaches the critical point, theoretically,

the system can be expressed in a very simple form, i.e., generally it can be expressed by fewervariables in an abstract phase space around a codimension-one bifurcation point, due to the bifurcation theory and center manifold theory. Thus, in the pre-disease state, we can express the system in a simple form to detect the signal. However, unfortunately, they are generally unobservable because of the abstract phase space. In our previous work, we constructed the relationship of the observable subnetwork (DNB) in the original state space with the unobservable variables of the abstract phase space, and we derived the conditions to identify the signal by only observing the DNB in the pre-disease state. Generally, we cannot derive the same conclusions for the normal state or the disease state. In addition, next, we provide several examples to numerically identify the DNB data when the system reaches the critical state according to the conclusions obtained.

B. Numerical simulations

To validate the reasonability of the statistical indicators obtained for critical transitions and detect early-warning signals near a tipping point, we numerically simulate three groups of real data for the complex diseases, such as H3N2, H1N1 and Lung.

Note that by the theoretical analysis above, we obtained some useful conclusions for biomarkers and nonbiomarkers. These conclusions just provides the criteria for us to identify biomarkers in the real data. Our idea is that if the behaviors of the proteins in each real data satisfy the obtained criteria, then we can view this protein as just the biomarker which we want to obtain.

Example 1. In this example, we take the real data for the complex disease H3N2. The simulation results for three indicators CV, TPC, TPD are presented in FiguresS1-S6, respectively.

FigureS1 shows the change of the coefficient of variation CV for 22 proteins: APOL6, CASP1, CNP, CXCL10,DHX58, DRAP1, DYNLT1, FAM46A, GEMIN4, GORASP1, IFITM1, IRF7, PLSCR1, RTP4, SAMD9, SAMHD1, SIGLEC1, TAF1C, TLR7, TNFAIP6,TREX1 and ZBP1. Figure S1 indicates that as the critical transition occurs, i.e., the time t evolves towards $t = 45h$ (see the vertical black line), the coefficient of variation CV for 22 proteins above significantly increases. That is, the behaviors of these proteins satisfy the corresponding criteria. Therefore, these 22 proteins can be viewed as DNBs for early warning signals.

Figures S2-S5show the change of the indicator $TPC = -\log(1-|PCC|)$ for 79protein pairs:

APOL6-CNP, APOL6-DRAP1, APOL6-DYNLT1, APOL6-GORASP1, APOL6-IFITM1, APOL6-PLSCR1, APOL6-SAMD9, APOL6-TNFAIP6, APOL6-TREX1, APOL6-ZBP1, CASP1-FAM46A, CASP1-GORASP1, CASP1-SAMHD1, CASP1-TLR7, CXCL10-DYNLT1, CXCL10-GORASP1, CXCL10-TNFAIP6, DHX58-GEMIN4, DHX58-GORASP1, DHX58-RTP4, DHX58-SAMD9, DHX58-SAMHD1, DHX58-SIGLEC1

DHX58-TLR7, DHX58-TNFAIP6, DHX58-ZBP1, DRAP1-GORASP1, DRAP1-SAMHD1, DRAP1-TLR7, DRAP1-TNFAIP6, DRAP1-ZBP1, DYNLT1-FAM46A, DYNLT1-PLSCR1, DYNLT1-RTP4, DYNLT1-SAMD9, DYNLT1-SIGLEC1, DYNLT1-TLR7, FAM46A-IFITM1, FAM46A-RTP4, FAM46A-SAMD9, FAM46A-SAMHD1, FAM46A-TLR7, FAM46A-TNFAIP6, GEMIN4-IFITM1, GEMIN4-PLSCR1, GEMIN4-SAMD9

GEMIN4-TNFAIP6, GORASP1-IRF7, GORASP1-PLSCR1, GORASP1-RTP4, GORASP1-ZBP1, IFITM1-PLSCR1, IFITM1-SAMD9, IFITM1-TLR7, IFITM1-TNFAIP6, IRF7-TLR7, IRF7-TNFAIP6, PLSCR1-RTP4, PLSCR1-TAF1C, PLSCR1-TLR7, PLSCR1-TNFAIP6,

PLSCR1-TREX1, PLSCR1-ZBP1, RTP4-SAMD9, RTP4-SAMHD1, RTP4-SIGLEC1, RTP4-TLR7, RTP4-ZBP1, SAMD9-SAMHD1

SAMD9-TNFAIP6, SAMD9-ZBP1, SAMHD1-TREX1, SIGLEC1-TLR7, SIGLEC1-TNFAIP6, SIGLEC1-ZBP1, TAF1C-TNFAIP6, TLR7-ZBP1, TNFAIP6-TREX1, TNFAIP6-ZBP1

These figures indicates that as the critical transition occurs, i.e., the time t evolves towards $t = 45h$ (see the vertical black line), the indicator TPC for 79 protein pairs significantly increases. That is, the behaviors of these proteins satisfy the corresponding criteria. Therefore, these 79 protein pairs can be viewed as biomarker pairs for early warning signals.

Figure S6 shows the change of the indicator TPD for 22 proteins: APOL6, CASP1, CNP, CXCL10,DHX58, DRAP1, DYNLT1, FAM46A, GEMIN4, GORASP1, IFITM1, IRF7,PLSCR1, RTP4, SAMD9, SAMHD1, SIGLEC1, TAF1C, TLR7, TNFAIP6,TREX1 and ZBP1. Figure S5 indicates that as the critical transition occurs, i.e., the time t evolves towards $t = 45h$ (see the vertical black line), the indicator TPD for 22 proteins above significantly increases. That is, the behaviors of these proteins satisfy the corresponding criteria. Therefore, these 22 proteins can be viewed as DNBs for early warning signals.

Example 2. In this example, we take the data for the complex disease H1N1. The simulation results for three indicators CV, TPC, TPD are presented in Figure.S7-S9, respectively.

Figure S7 shows the change of the coefficient of variation CV for 20 proteins:ACP6, BTG1, CCNA1, DDX18, DKC1, H2AFV, HMGN1, IRF7, ITK, LAX1, NPAT, NR2C1, PFN2, POLR1C, RBM4B, RPS2, SLBP, SP110, STMN1 and VPRBP. Figure S7 indicates that as the critical transition occurs, i.e., the time t evolves towards $t = 53h$ (see the vertical black line), the coefficient of variation CV and the indicator TPD for 20 proteins above significantly increases. That is, the behaviors of these proteins satisfy the corresponding criteria. Therefore, these 20 proteins can be viewed as DNBs for early warning signals.

Figure S8 also shows the change of the indicator $TPC = -\log\left(1-\left|PCC\right|\right)$ for 16 protein pairs:ACP6-DKC1, ACP6-ITK, ACP6-NPAT, ACP6-STMN1, BTG1-CCNA1, DDX18-NUCB1, DKC1-NR2C1, H2AFV-SLBP, HMGN1-ITK, HMGN1-PFN2, HMGN1-SEPT4, IRF7-SP110, LAX1-VPRBP, POLR1C-STMN1, RBM4B-RPS2, and SEPT4-STMN1.Figure S8 indicates that as the critical transition occurs, i.e., the time t evolves towards $t = 53h$ (see the vertical black line), the indicator TPC for 16 protein pairs significantly increases. That is, the behaviors of these proteins satisfy the corresponding criteria. Therefore, these 16 protein pairs can be viewed as biomarker pairs for early warning signals.

Figure S9 shows the change of the indicator TPD for 3 proteins: CCNA1, NUCB1 and SEPT4. Figure S9 indicates that as the critical transition occurs, i.e., the time t evolves towards $t = 53h$ (see the vertical black line), the indicator TPD for 3 proteins above significantly increases. That is, the behaviors of these proteins satisfy the corresponding criteria. Therefore, these 3 proteins can be viewed as DNBs for early warning signals.

Example 3. In this example, we take the real data for the complex disease Lung. The simulation results for three indicators CV, TPC, TPD are presented in Figures.S10-S17, respectively.

Figure S10 shows the change of the coefficient of variation CV for 18 proteins: Actn1, Adcy8, Atp6v1d, Capn1, Clstn1, Dapk1, Fzd2, Gnb1, H1f0, Hhip, Hprt1, Htra1, Kcnq1, Nagk, Rad17, Rad23b, Tjp2 and Wbp1. Figure S10 indicates that as the critical transition occurs, i.e., the time t evolves towards $t = 8h$ (see the vertical black line), the coefficient of variation CV for 18 proteins above significantly increases. That is, the behaviors of these proteins satisfy the corresponding criteria. Therefore, these 18 proteins can be viewed as DNBs for early warning signals.

Figures S11-S15show the change of the indicator $TPC = -\log\left(1-\left|PCC\right|\right)$ for 112 protein pairs:

Abcd3-Mcee, Actn1-Ereg, Actn1-Nr2f6, Actn1-Psmd4, Adcy8-Adss,
Adss-Adcy8, Adss-Ensmusg00000050347, Adss-Gp49a, Anxa1-Ddx39, Anxa1-Fzd2,
Aplp2-Aqp1, Aplp2-Fzd2, Aplp2-Kcnq1, Aqp1-Aplp2, Aqp1-Nagk,
Aqp1-Psmd4, Atp6v1d-Prpf40a, Capn1-Clstn1, Capn1-Glrx, Clstn1-Capn1,
Clstn1-Psma7, Csf1r-Htra1, Csf1r-Psmc3

Dapk1-Gp49a, Dapk1-Klhl13, Ddx39-Anxa1, Ddx39-Kcnq1, Ensmusg00000050347-Adss,
Ereg-Actn1, Ereg-Hhip, Ereg-Mmp19, Ereg-Pla2g15, Ereg-Psme4,
Faf1-Ulk2, Fzd2-Anxa1, Fzd2-Aplp2, Fzd2-Stxbp1, G6pd2-Nagk,
Gimap4-Lox, Glrx-Capn1, Glrx-Sin3b, Gnb1-H1f0, Gnb1-Mmp19,
Gp49a-Adss, Gp49a-Dapk1, Grem2-Psma1,

Gtf2i-Hnrnpd, H1f0-Gnb1, H1f0-Hnrnpd, H1f0-Rad23b, Hhip-Ereg,
Hist2h2bb-Psmd13, Hnrnpd-Gtf2i, Hnrnpd-H1f0, Hnrnpd-Klhl13, Hnrnpd-Rad23b,
Hprt1-Ncl, Hprt1-Nr2f6, Htra1-Csf1r, Htra1-Prelp, Kcnq1-Aplp2,
Kcnq1-Ddx39, Klhl13-Dapk1, Klhl13-Hnrnpd, Lox-Gimap4, Lrg1-Wbp1,
Macf1-Nrp1, Macf1-Psmd4, Mcee-Abcd3

Mmp19-Ereg, Mmp19-Gnb1, Mmp19-Rad23b, Mmp19-Thbs3, Nagk-Aqp1,
Nagk-G6pd2, Ncl-Hprt1, Nr2f6-Actn1, Nr2f6-Hprt1, Nr2f6-Psma7,
Nrp1-Macf1, Phlpp-Psma1, Pla2g15-Ereg, Prelp-Htra1, Prpf40a-Atp6v1d,
Psma1-Grem2, Psma1-Phlpp, Psma7-Clstn1, Psma7-Nr2f6, Psmb5-Psmd4,
Psmc3-Csf1r, Psmd13-Hist2h2bb, Psmd4-Actn1

Psmd4-Aqp1, Psmd4-Macf1, Psmd4-Psmb5, Psmd4-Rad17, Psmd4-Rad23b,
Psmd4-Tjp2, Psme4-Ereg, Rad17-Psmd4, Rad23b-H1f0, Rad23b-Hnrnpd,
Rad23b-Mmp19, Rad23b-Psmd4, Sin3b-Glrx, Stxbp1-Fzd2, Stxbp1-Wbp1,
Thbs3-Mmp19, Tjp2-Psmd4, Ulk2-Faf1, Wbp1-Lrg1, Wbp1-Stxbp1

Figures S11-S15 indicate that as the critical transition occurs, i.e., the time t evolves towards $t = 8h$ (see the vertical black line), the indicator TPC for 112 protein pairs significantly increases. That is, the behaviors of these proteins satisfy the corresponding criteria. Therefore, these 112 protein pairs can be viewed as biomarker pairs for early warning signals.

Figures S16 and S17 show the change of the indicator TPD for 42 proteins: Abcd3, Adss, Anxa1, Aplp2, Aqp1, Csf1r, Ddx39, Ensmusg00000050347, Ereg, Faf1, G6pd2, Gimap4, Glrx, Gp49a, Grem2, Gtf2i, Hist2h2bb, Hnrnpd, Klhl13, Lox, Lrg1, Macf1, Mcee, Mmp19, Ncl, Nr2f6, Nrp1, Phlpp, Pla2g15, Prelp, Prpf40a, Psma1, Psma7, Psmb5, Psmc3, Psmd13, Psmd4, Psme4, Sin3b, Stxbp1, Thbs3 andUlk2. Figures S16 and S17 indicate that as the critical transition occurs, i.e., the time t evolves towards $t = 8h$ (see the vertical black line), the indicator TPD for 42 proteins above significantly increases. That is, the

behaviors of these proteins satisfy the corresponding criteria. Therefore, these 42 proteins can be viewed as DNBs for early warning signals.

In summary, it is worthy pointing out something about choosing biomarkers in algorithms. For H3N2, only for the indicators CV or TPD, we can choose 264 proteins and 116 proteins, respectively; then taking the intersection of both of them, we continue to choose 22 proteins from the obtained intersection proteins, for each of which there exists another one from these 22 proteins such that the indicator TPC increases significantly as the time t evolves towards $t = 45h$ (see the vertical black line). In this way, we obtain the DNBs.

However, for the complex disease H1N1 and Lung, we choose DNBs in another way. Here, we take H1N1 as an example. Firstly, only for the indicators CV or TPD, we can choose 32 proteins and 6 proteins, respectively; but the intersection of them is few(maybe the result is empty set). Then, the numerical simulation for the indicator TPC cannot be done. Due to this reason, we take the union of the chosen proteins for CV and proteins for TPD. From the union proteins, we continue to choose sevaral protein pairs, for which the indicator TPC increases significantly as the time evolves towards $t = 8h$ (see the vertical black line). From these chosen protein pairs, we obtain the DNBs.

In our opinion, provided that one of the indicators CV and TPD changes significantly, we can think the corresponding protein is a biomarker candidate, because the increasing tendency of CV means that the concentration of the protein fluctuates more strongly, and the increasing tendency of TPD means that the probability distribution of the concentration of the protein changes significantly. Intuitively, it is easy to understand that the critical transition will occur. Therefore, the numerical method for the complex disease H1N1 is proper. Since our purpose of doing numerical simulations is just to verify the validity of the conclusions obtained, we can know that the numerical methods mentioned above make sense.

C.Supplementary Figures

C1. The dataset for H3N2.

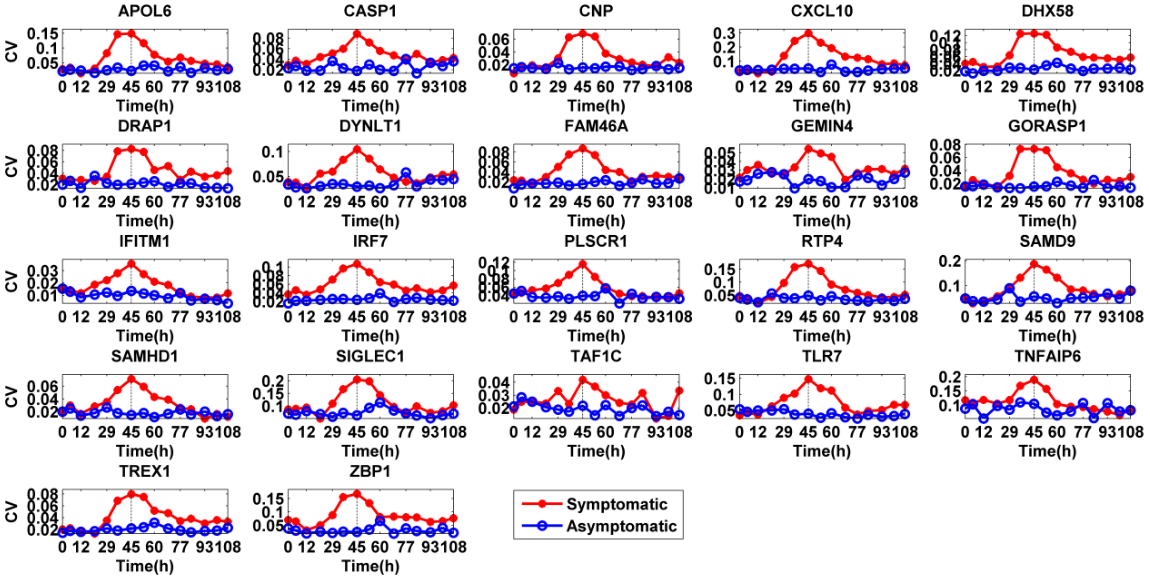

Figure.S1: The change of the coefficient of variation CV for 22 proteins for H3N2: APOL6, CASP1, CNP, CXCL10, DHX58, DRAP1, DYNLT1, FAM46A, GEMIN4, GORASP1, IFITM1,IRF7, PLSCR1, RTP4, SAMD9, SAMHD1, SIGLEC1, TAF1C, TLR7, TNFAIP6, TREX1 and ZBP1. It indicates that as the critical transition occurs, i.e., the time evolves towards $t = 45\text{h}$ (see the vertical black line), the coefficient of variation CV for 22 proteins above significantly increases in the symptomatic group and has no obvious change in the asymptomatic group. That is, the behaviors of these proteins satisfy the corresponding criteria. Therefore, these 22 proteins can be viewed as DNBs for early warning signals.

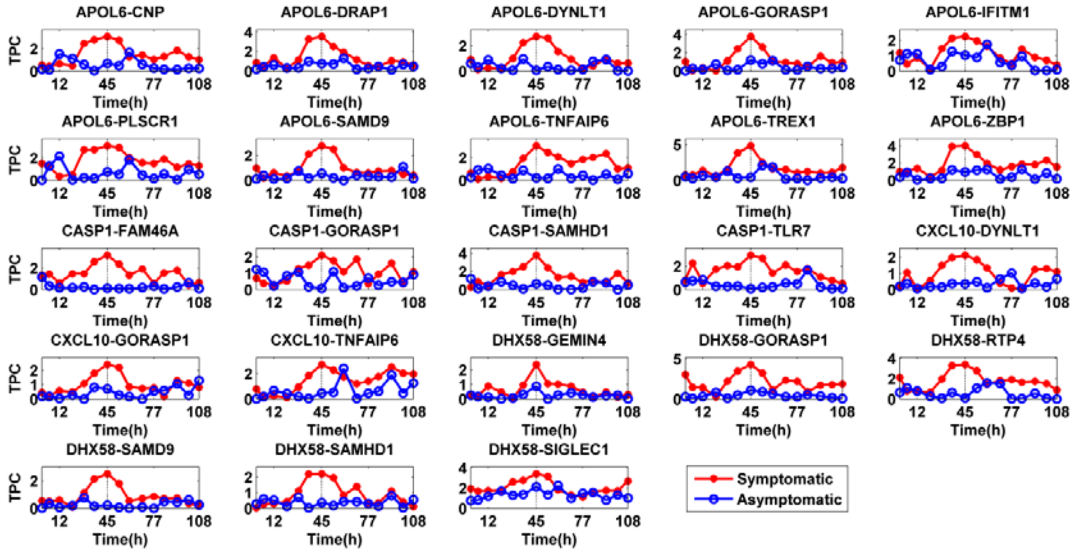

Figure.S2: The change of the indicator TPC for 23 protein pairs: APOL6-CNP, APOL6-DRAP1, APOL6-DYNLT1, APOL6-GORASP1, APOL6-IFITM1, APOL6-PLSCR1,APOL6-SAMD9, APOL6-TNFAIP6, APOL6-TREX1, APOL6-ZBP1, CASP1-FAM46A, CASP1-GORASP1, CASP1-SAMHD1, CASP1-TLR7, CXCL10-DYNLT1, CXCL10-GORASP1, CXCL10-TNFAIP6, DHX58-GEMIN4, DHX58-GORASP1, DHX58-RTP4, DHX58-SAMD9, DHX58-SAMHD1, and DHX58-SIGLEC1.

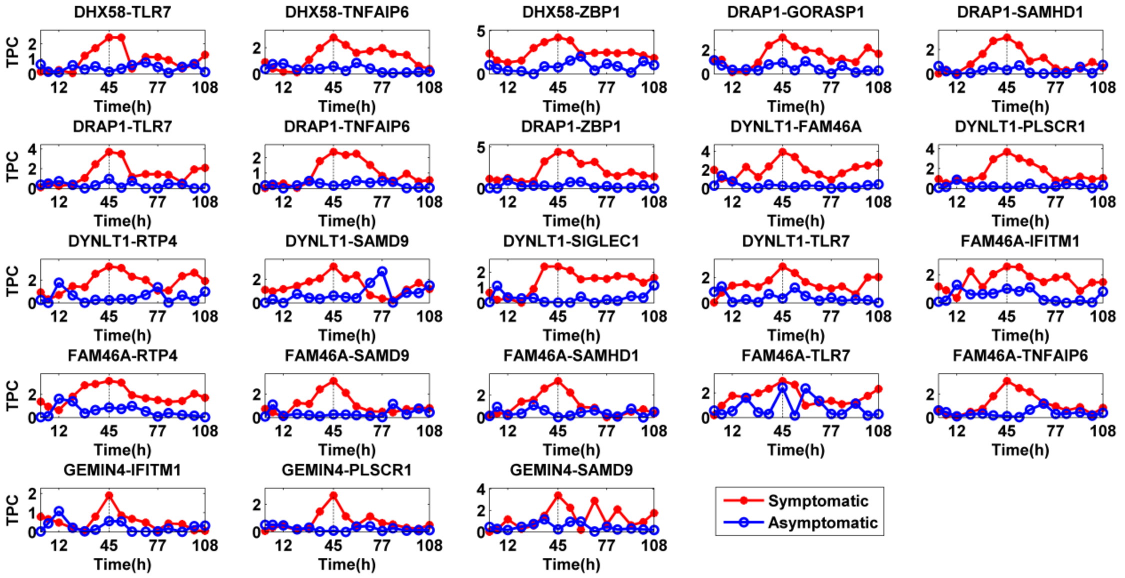

Figure.S3: The change of the indicator TPC for 23 protein pairs: DHX58-TLR7, DHX58-TNFAIP6, DHX58-ZBP1, DRAP1-GORASP1, DRAP1-SAMHD1, DRAP1-TLR7, DRAP1-TNFAIP6, DRAP1-ZBP1, DYNLT1-FAM46A, DYNLT1-PLSCR1, DYNLT1-RTP4, DYNLT1-SAMD9, DYNLT1-SIGLEC1, DYNLT1-TLR7, FAM46A-IFITM1, FAM46A-RTP4, FAM46A-SAMD9, FAM46A-SAMHD1, FAM46A-TLR7, FAM46A-TNFAIP6, GEMIN4-IFITM1, GEMIN4-PLSCR1, and GEMIN4-SAMD9.

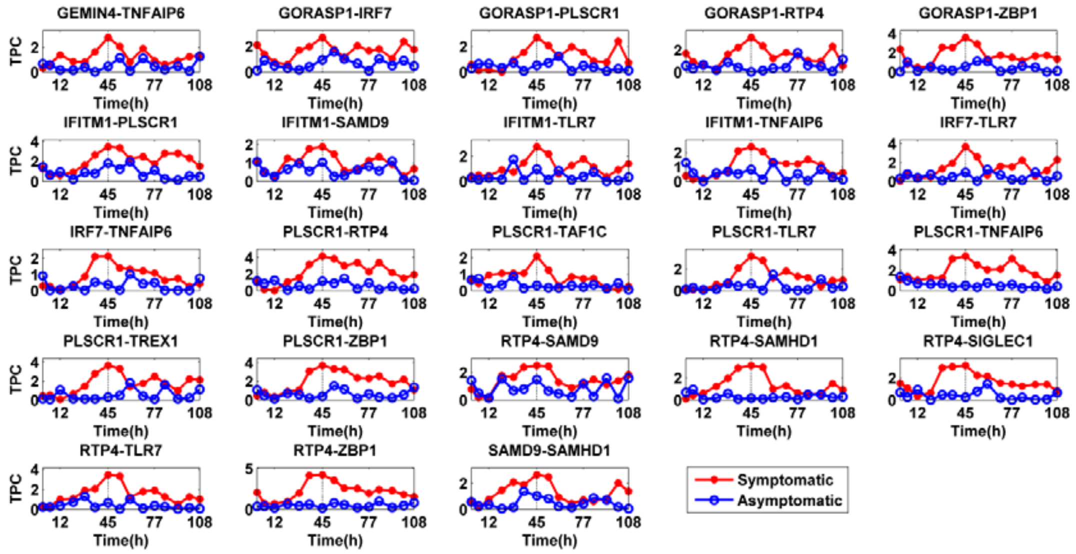

Figure.S4: The change of the indicator TPC for 23 protein pairs: GEMIN4-TNFAIP6, GORASP1-IRF7, GORASP1-PLSCR1, GORASP1-RTP4, GORASP1-ZBP1, IFITM1-PLSCR1, IFITM1-SAMD9, IFITM1-TLR7, IFITM1-TNFAIP6, IRF7-TLR7, IRF7-TNFAIP6, PLSCR1-RTP4, PLSCR1-TAF1C, PLSCR1-TLR7, PLSCR1-TNFAIP6, PLSCR1-TREX1, PLSCR1-ZBP1, RTP4-SAMD9, RTP4-SAMHD1, RTP4-SIGLEC1, RTP4-TLR7, RTP4-ZBP1, and SAMD9-SAMHD1.

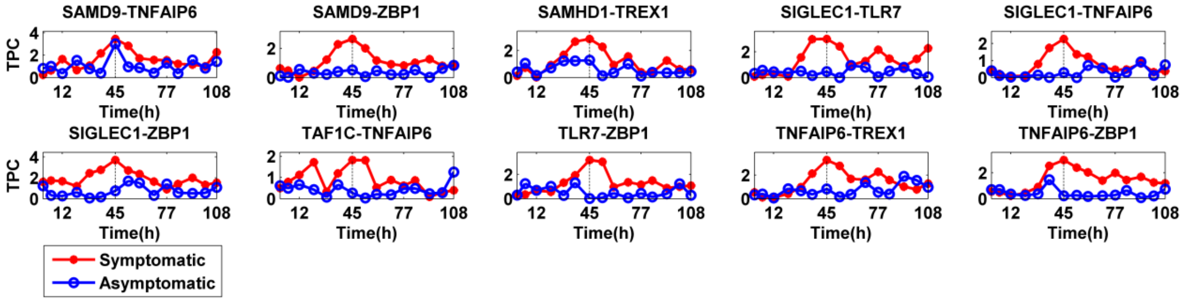

Figure.S5: The change of the indicator TPC for 10 protein pairs: SAMD9-TNFAIP6, SAMD9-ZBP1, SAMHD1-TREX1, SIGLEC1-TLR7, SIGLEC1-TNFAIP6, SIGLEC1-ZBP1, TAF1C-TNFAIP6, TLR7-ZBP1, TNFAIP6-TREX1, and TNFAIP6-ZBP1.

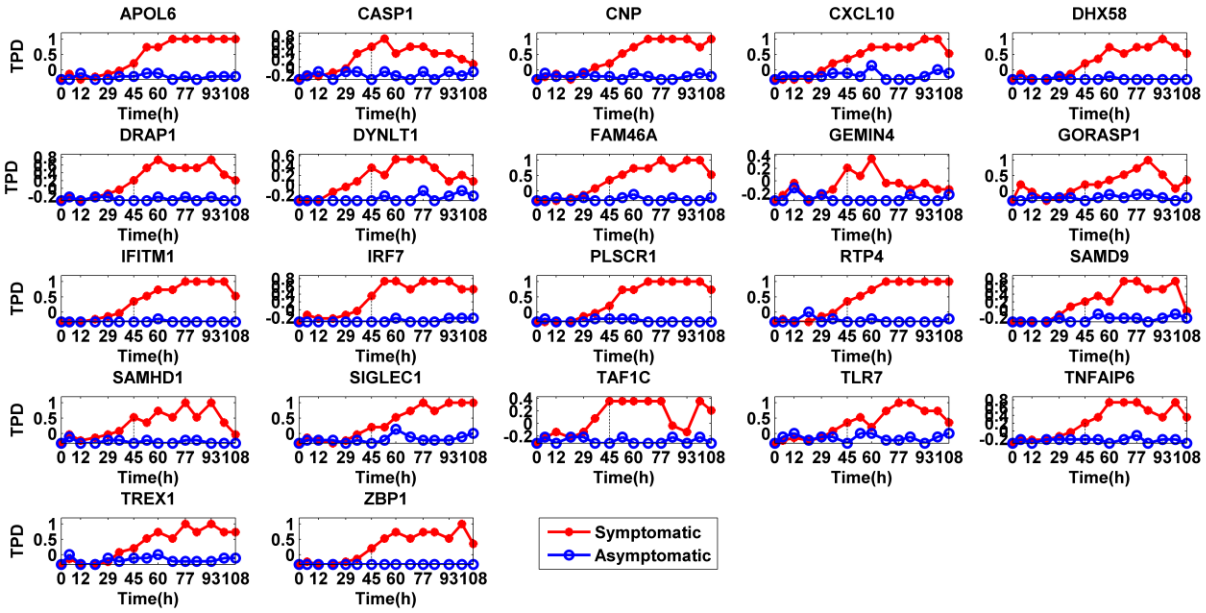

Figure.S6: The change of the indicator TPD for 22 proteins: APOL6, CASP1, CNP, CXCL10, DHX58, DRAP1, DYNLT1, FAM46A, GEMIN4, GORASP1, IFITM1, IRF7,PLSCR1, RTP4, SAMD9, SAMHD1, SIGLEC1, TAF1C, TLR7, TNFAIP6, TREX1 and ZBP1. The x-axis denotes the time(unit: h). It indicates that as the critical transition occurs,i.e., the time evolves towards $t = 45h$ (see the vertical black line), the indicator TPD for 22 proteins above significantly increases. That is, the behaviors of these proteins satisfy the corresponding criteria. Therefore, these 22 proteins can be viewed as DNBs for early warning signals.

C2 The dataset for H1N1.

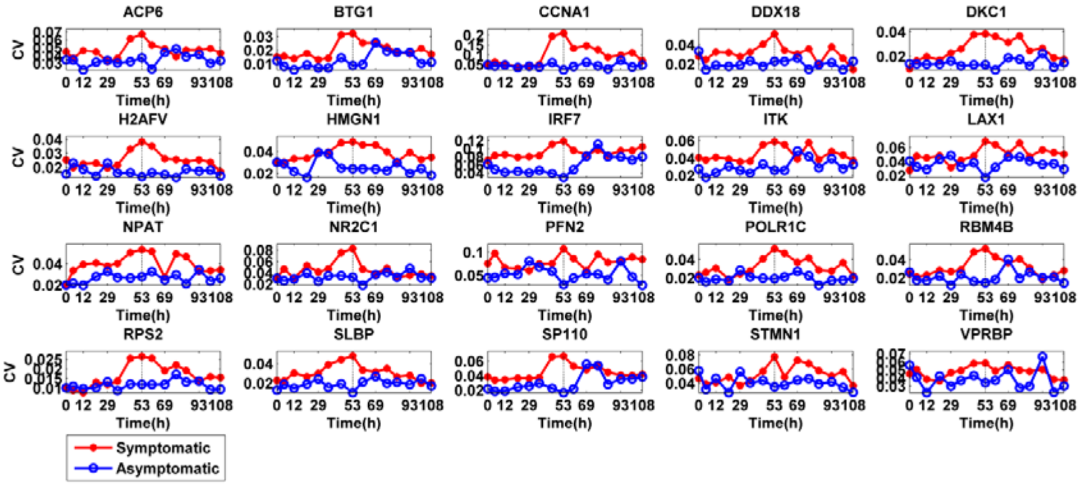

Figure.S7. The change of the coefficient of variation CV for 20 proteins: ACP6, BTG1, CCNA1, DDX18, DKC1, H2AFV, HMGN1, IRF7, ITK, LAX1, NPAT, NR2C1, PFN2, POLR1C, RBM4B, RPS2, SLBP, SP110, STMN1 and VPRBP. The x-axis denotes the time(unit: h). It indicates that as the critical transition

occurs, i.e., the time evolves towards $t = 53h$ (see the vertical black line), the coefficient of variation CV for 20 proteins above significantly increases in the symptomatic group and has no obvious change in the asymptomatic group. That is, the behaviors of these proteins satisfy the corresponding criteria. Therefore, these 20 proteins can be viewed as DNBs for early warning signals.

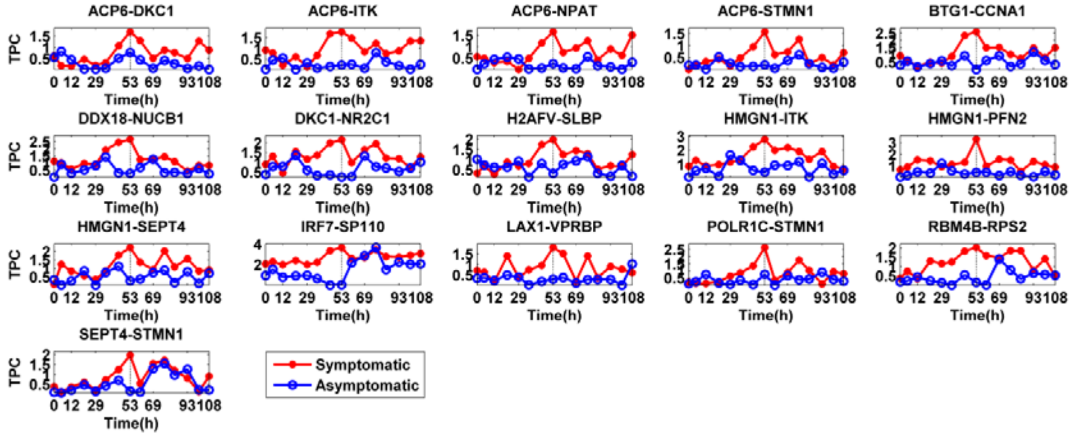

Figure.S8: The change of the indicator TPC for 16 protein pairs: ACP6-DKC1, ACP6-ITK, ACP6-NPAT, ACP6-STMN1, BTG1-CCNA1, DDX18-NUCB1, DKC1-NR2C1, H2AFV-SLBP, HMGN1-ITK, HMGN1-PFN2, HMGN1-SEPT4, IRF7-SP110, LAX1-VPRBP, POLR1C-STMN1, RBM4B-RPS2, and SEPT4-STMN1. The x-axis denotes the time(unit: h).

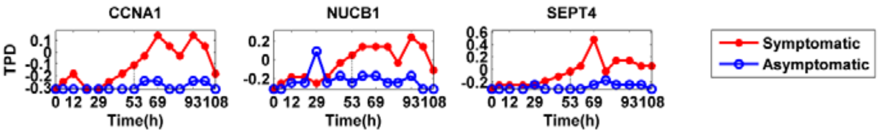

Figure.S9. The change of the indicator TPD for 3 proteins: CCNA1, NUCB1 and SEPT4. The x-axis denotes the time(unit: h). It indicates that as the critical transition occurs, i.e., the time evolves towards $t = 53h$ (see the vertical black line), the indicator TPD for 3 proteins above significantly increases. That is, the behaviors of these proteins satisfy the corresponding criteria. Therefore, these 3 proteins can be viewed as DNBs for early warning signals.

C3 The data for acute lung injury

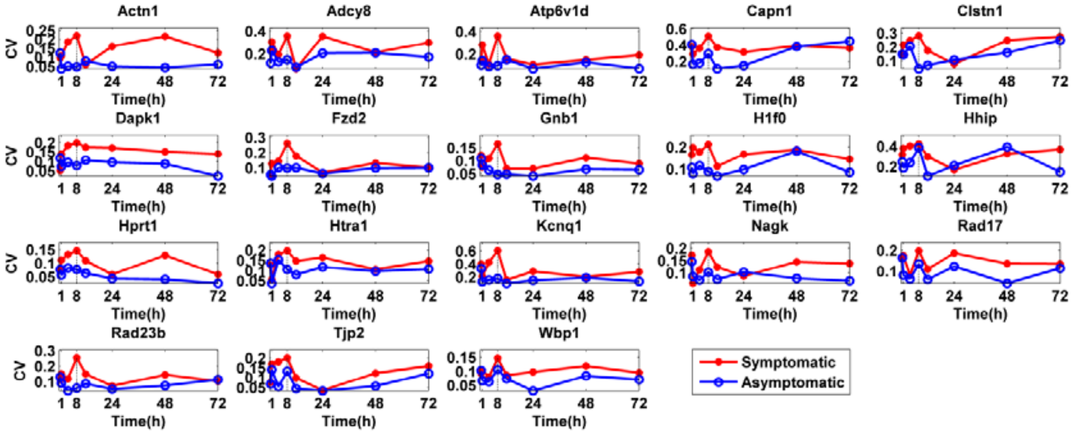

Figure.S10: The change of the coefficient of variation CV for 18 proteins: Actn1, Adcy8, Atp6v1d, Capn1, Clstn1, Dapk1, Fzd2, Gnb1, H1f0, Hhip, Hprt1, Htra1, Kcnq1, Nagk, Rad17, Rad23b, Tjp2 and Wbp1. It indicates that as the critical transition occurs, i.e., the time evolves towards $\mathrm{t} = 8\mathrm{h}$ (see the vertical black line), the coefficient of variation CV for 18 proteins above significantly increases in the symptomatic group and has no obvious change in the asymptomatic group. That is, the behaviors of these proteins satisfy the corresponding criteria. Therefore, these 18 proteins can be viewed as DNBs for early warning signals.

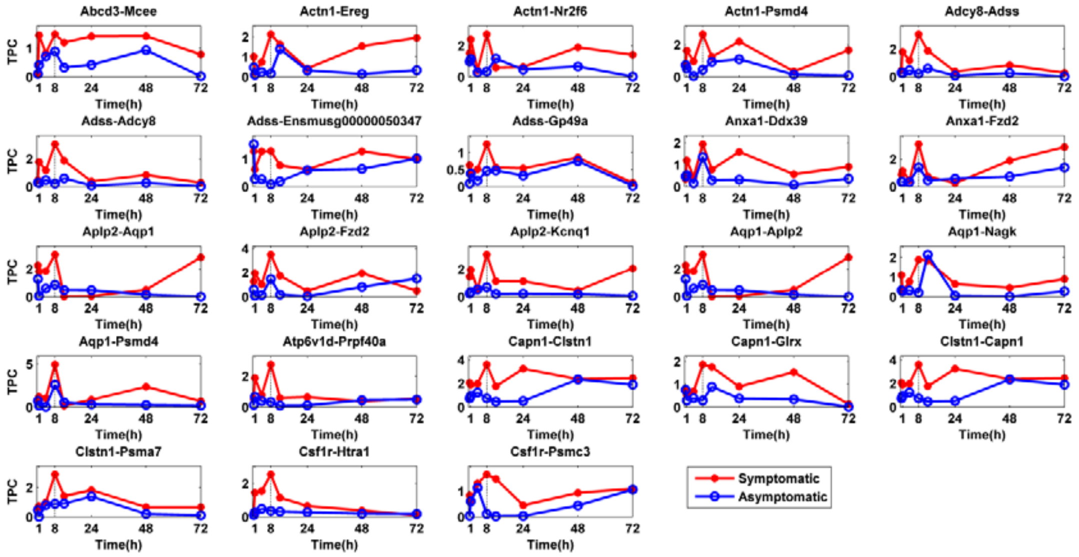

Figure.S11: The change of the indicator TPC for 23 protein pairs: Abcd3-Mcee, Actn1-Ereg, Actn1-Nr2f6, Actn1-Psmd4, Adcy8-Adss, Adss-Adcy8, Adss-Ensmusg00000050347, Adss-Gp49a, Anxa1-Ddx39, Anxa1-Fzd2, Aplp2-Aqp1, Aplp2-Fzd2, Aplp2-Kcnq1, Aqp1-Aplp2, Aqp1-Nagk, Aqp1-Psmd4, Atp6v1d-Prpf40a, Capn1-Clstn1, Capn1-Glrx, Clstn1-Capn1, Clstn1-Psma7, Csf1r-Htra1, and Csf1r-Psmc3. The x-axis denotes the time(unit: h).

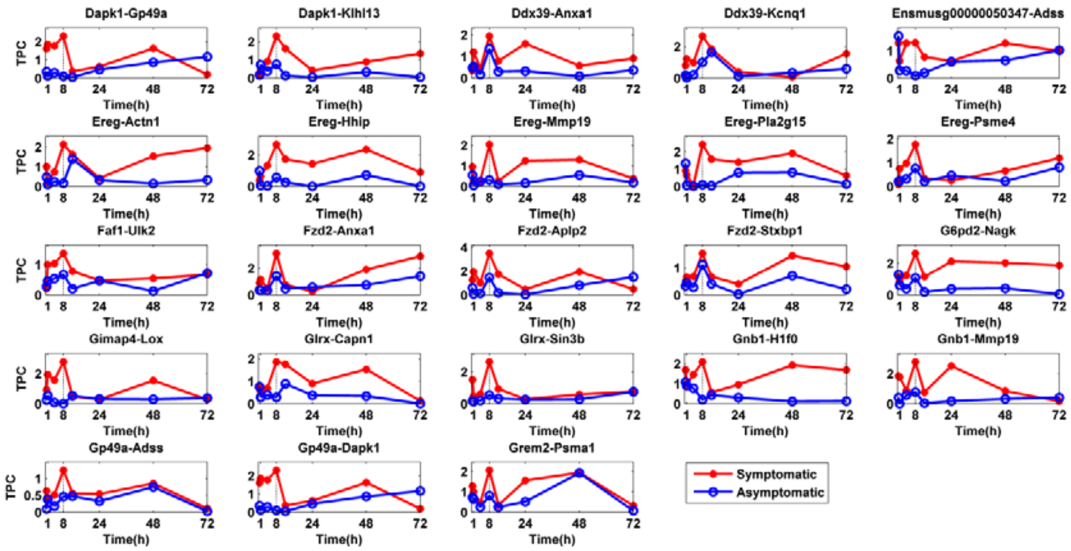

Figure.S12: The change of the indicator TPC for 23 protein pairs: Dapk1-Gp49a, Dapk1-Klhl13, Ddx39-Anxa1, Ddx39-Kcnq1, Ensmusg00000050347-Adss, Ereg-Actn1, Ereg-Hhip, Ereg-Mmp19, Ereg-Pla2g15, Ereg-Psme4, Faf1-Ulk2, Fzd2-Anxa1, Fzd2-Aplp2, Fzd2-Stxbp1, G6pd2-Nagk, Gimap4-Lox, Glrx-Capn1, Glrx-Sin3b, Gnb1-H1f0, Gnb1-Mmp19, Gp49a-Adss, Gp49a-Dapk1, and Grem2-Psma1. The x-axis denotes the time(unit: h).

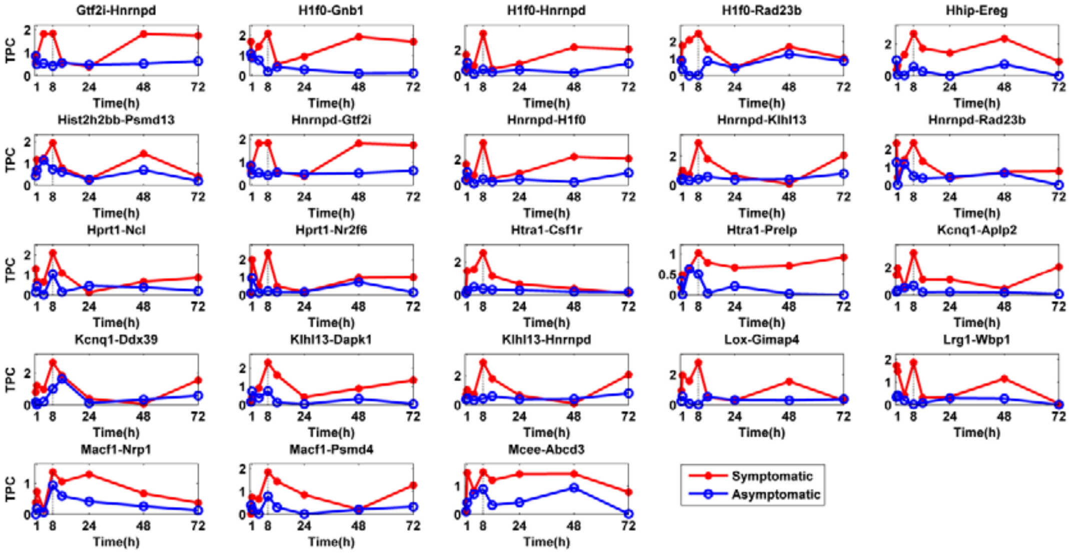

Figure.S13: The change of the indicator TPC TPC for 23 protein pairs: Gtf2i-Hnrnpd, H1f0-Gnb1, H1f0-Hnrnpd, H1f0-Rad23b, Hhip-Ereg, Hist2h2bb-Psmd13, Hnrnpd-Gtf2i, Hnrnpd-H1f0, Hnrnpd-Klhl13, Hnrnpd-Rad23b, Hprt1-Ncl, Hprt1-Nr2f6, Htra1-Csf1r, Htra1-Prelp, Kcnq1-Aplp2, Kcnq1-Ddx39, Klhl13-Dapk1, Klhl13-Hnrnpd, Lox-Gimap4, Lrg1-Wbp1, Macf1-Nrp1, Macf1-Psmd4, and Mcee-Abcd3. The x-axis denotes the time(unit: h).

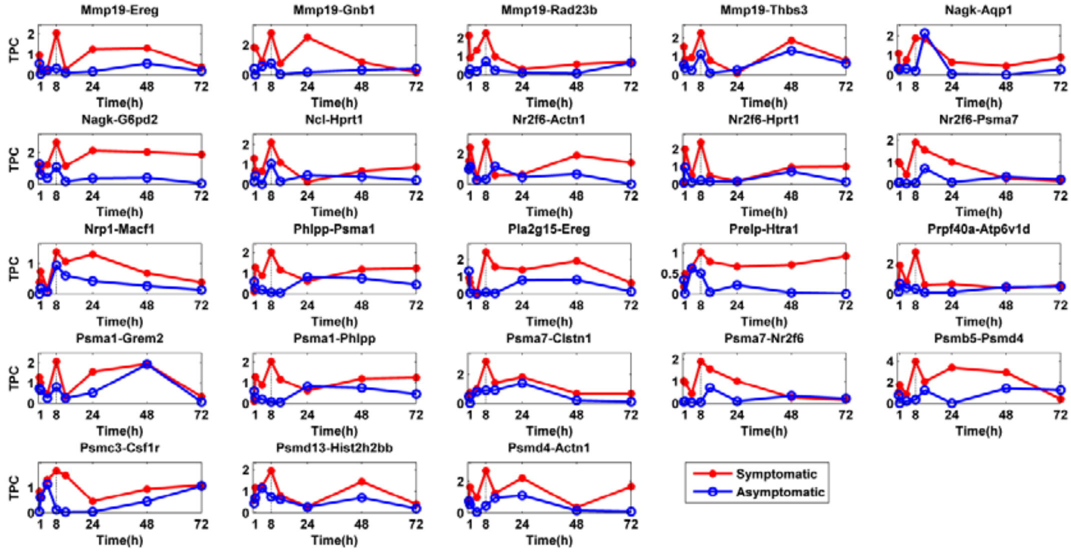

Figure.S14: The change of the indicator TPC TPC for 23 protein pairs: Mmp19-Ereg, Mmp19-Gnb1, Mmp19-Rad23b, Mmp19-Thbs3, Nagk-Aqp1, Nagk-G6pd2, Ncl-Hprt1, Nr2f6-Actn1, Nr2f6-Hprt1,

Nr2f6-Psma7, Nrp1-Macf1, Phlpp-Psma1, Pla2g15-Ereg, Prelp-Htra1, Prpf40a-Atp6v1d, Psma1-Grem2, Psma1-Phlpp, Psma7-Clstn1, Psma7-Nr2f6, Psmb5-Psmd4, Psmc3-Csf1r, Psmd13-Hist2h2bb, and Psmd4-Actn1.

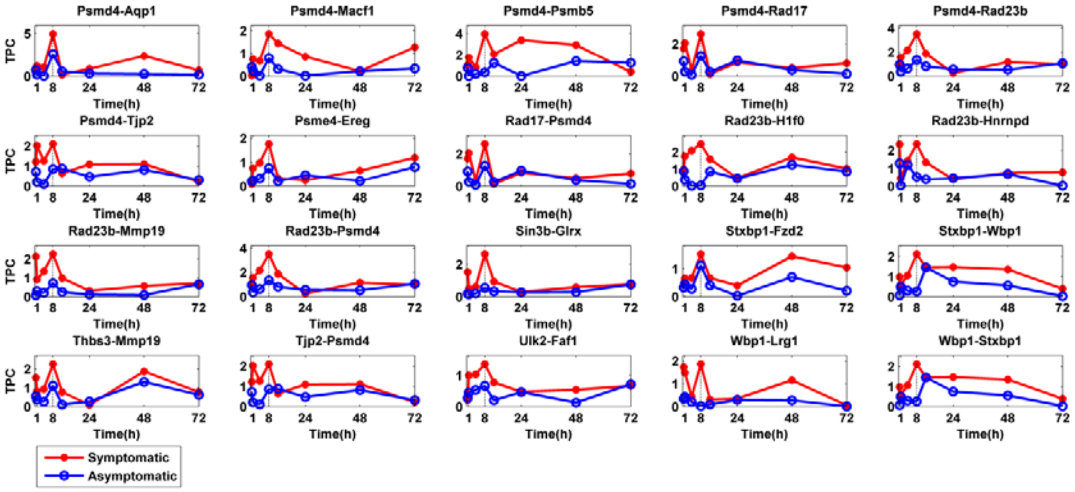

Figure.S15: The change of the indicator TPC TPC for 20 protein pairs: Psmd4-Aqp1, Psmd4-Macf1, Psmd4-Psmb5, Psmd4-Rad17, Psmd4-Rad23b, Psmd4-Tjp2, Psme4-Ereg, Rad17-Psmd4, Rad23b-H1f0, Rad23b-Hnrnpd, Rad23b-Mmp19, Rad23b-Psmd4, Sin3b-Glrx, Stxbp1-Fzd2, Stxbp1-Wbp1, Thbs3-Mmp19, Tjp2-Psmd4, Ulk2-Faf1, Wbp1-Lrg1, and Wbp1-Stxbp1. The x-axis denotes the time(unit: h).

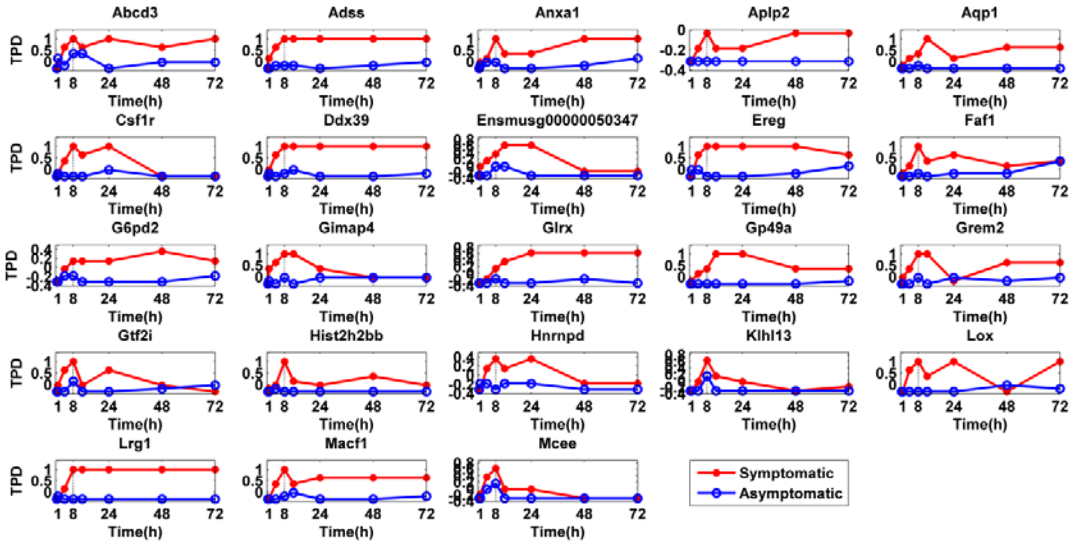

Figure.S16: The change of the indicator TPD for 23 proteins: Abcd3, Adss, Anxa1, Aplp2, Aqp1, Csf1r, Ddx39, Ensmusg00000050347, Ereg, Faf1, G6pd2, Gimap4, Glrx, Gp49a, Grem2, Gtf2i, Hist2h2bb, Hnrnpd, Klhl13, Lox, Lrg1, Macf1 and Mcee. It indicates that as the critical transition occurs, i.e., the time evolves towards $t = 8h$ (see the vertical black line), the indicator TPD for 23 proteins above significantly increases. That is, the behaviors of these proteins satisfy the corresponding criteria. Therefore, these 23 proteins can be viewed as DNBs for early warning signals.

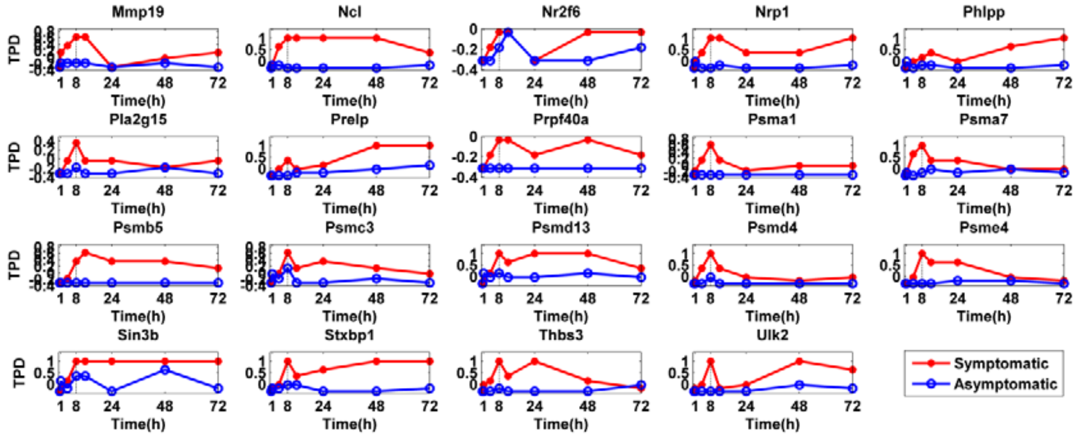

Figure.S17: The change of the indicator *TPD* for 19 proteins: Mmp19, Ncl, Nr2f6, Nrp1, Phlpp, Pla2g15, Prelp, Prpf40a, Psma1, Psma7, Psmb5, Psmc3, Psmd13, Psmd4, Psme4, Sin3b, Stxbp1, Thbs3 and Ulk2. The x-axis denotes the time(unit: h). It indicates that as the critical transition occurs, i.e., the time evolves towards $t = 8$h (see the vertical black line), the indicator *TPD* for 19 proteins above significantly increases. That is, the behaviors of these proteins satisfy the corresponding criteria. Therefore, these 19 proteins can be viewed as DNBs for early warning signals.

References

Arnol, V.I. (1994) *Dynamical Systems V: Bifurcation theory and Catastrophe theory*.

Chen, L., Wang, R.-S. and Zhang, X.-S. (2009) *Biomolecular networks: methods and applications in systems biology*. John Wiley & Sons.

Chen, L., *et al.* (2010) *Modeling biomolecular networks in cells: structures and dynamics*. Springer Science & Business Media.

Chisholm, R.A. and Filotas, E. (2009) Critical slowing down as an indicator of transitions in two-species models, *Journal of theoretical biology*, **257**, 142-149.

Dahlem, M.A., *et al.* (2013) Towards dynamical network biomarkers in neuromodulation of episodic migraine, *Translational neuroscience*, **4**, 282-294.

Dai, L., Korolev, K.S. and Gore, J. (2013) Slower recovery in space before collapse of connected populations, *Nature*, **496**, 355-358.

Dai, L., Korolev, K.S. and Gore, J. (2015) Relation between stability and resilience determines the performance of early warning signals under different environmental drivers, *Proceedings of the National Academy of Sciences*, **112**, 10056-10061.

Dakos, V., *et al.* (2012) Methods for detecting early warnings of critical transitions in time series illustrated using simulated ecological data, *PloS one*, **7**, e41010.

Dakos, V., *et al.* (2010) Spatial correlation as leading indicator of catastrophic shifts, *Theoretical Ecology*, **3**, 163-174.

Drake, J.M. and Griffen, B.D. (2010) Early warning signals of extinction in deteriorating environments, *Nature*, **467**, 456-459.

Eikenberry, S., *et al.* (2009) The dynamics of a delay model of hepatitis B virus infection with logistic hepatocyte growth, *Mathematical Biosciences and Engineering*, **6**, 283-299.

Gao, J., Barzel, B. and Barabási, A.-L. (2016) Universal resilience patterns in complex networks, *Nature*, **530**, 307-312.
Guckenheimer, J. and Holmes, P. (1984) Nonlinear oscillations, dynamical systems and bifurcations of vector fields, *J. Appl. Mech*, **51**.
Hart, Y., *et al.* (2012) Design principles of cell circuits with paradoxical components, *Proceedings of the National Academy of Sciences*, **109**, 8346-8351.
Hirata, Y., Bruchovsky, N. and Aihara, K. (2010) Development of a mathematical model that predicts the outcome of hormone therapy for prostate cancer, *Journal of Theoretical Biology*, **264**, 517-527.
Hovinen, E., Kekki, M. and Kuikka, S. (1976) A theory to the stochastic dynamic model building for chronic progressive disease processes with an application to chronic gastritis, *Journal of theoretical biology*, **57**, 131-152.
Huang, S., Ernberg, I. and Kauffman, S. (2009) Cancer attractors: a systems view of tumors from a gene network dynamics and developmental perspective. *Seminars in cell & developmental biology*. Elsevier, pp. 869-876.
Kuehn, C. (2011) A mathematical framework for critical transitions: Bifurcations, fast–slow systems and stochastic dynamics, *Physica D: Nonlinear Phenomena*, **240**, 1020-1035.
Kuehn, C. (2013) A mathematical framework for critical transitions: normal forms, variance and applications, *Journal of nonlinear science*, **23**, 457-510.
Kuznetsov, Y.A. (2013) *Elements of applied bifurcation theory*. Springer Science & Business Media.
Lade, S.J. and Gross, T. (2012) Early warning signals for critical transitions: a generalized modeling approach, *PLoS Comput Biol*, **8**, e1002360.
Mao, X. (2007) *Stochastic differential equations and applications*. Elsevier.
Murdock, J. (2006) *Normal forms and unfoldings for local dynamical systems*. Springer Science & Business Media.
Puu, T. (2013) *Attractors, bifurcations, & chaos: Nonlinear phenomena in economics*. Springer Science & Business Media.
Scheffer, M., *et al.* (2009) Early-warning signals for critical transitions, *Nature*, **461**, 53-59.
Scheffer, M., *et al.* (2001) Catastrophic shifts in ecosystems, *Nature*, **413**, 591-596.
Scheffer, M., *et al.* (2015) Generic indicators of ecological resilience: inferring the chance of a critical transition, *Annual Review of Ecology, Evolution, and Systematics*, **46**, 145-167.
Tanaka, G., *et al.* (2008) Bifurcation analysis on a hybrid systems model of intermittent hormonal therapy for prostate cancer, *Physica D: Nonlinear Phenomena*, **237**, 2616-2627.
Van Nes, E.H. and Scheffer, M. (2007) Slow recovery from perturbations as a generic indicator of a nearby catastrophic shift, *The American Naturalist*, **169**, 738-747.
Veraart, A.J., *et al.* (2012) Recovery rates reflect distance to a tipping point in a living system, *Nature*, **481**, 357-359.
Voit, E.O. (2009) A systems-theoretical framework for health and disease: inflammation and preconditioning from an abstract modeling point of view, *Mathematical biosciences*, **217**, 11-18.
Wiggins, S. (1990) Global bifurcations and chaos: Analytical methods, volume 73 of Appl, *Math. Sci. Springer, New York*.
